# Vortex reversal is a precursor of confined bacterial turbulence

**DOI:** 10.1073/pnas.2414446122

**Published:** 2025-03-14

**Authors:** Daiki Nishiguchi, Sora Shiratani, Kazumasa A. Takeuchi, Igor S. Aranson

**Affiliations:** ^a^Department of Physics, School of Science, Institute of Science Tokyo, Meguro-ku, Tokyo 152–8551, Japan; ^b^Department of Physics, School of Science, The University of Tokyo, Bunkyo-ku, Tokyo 113–0033, Japan; ^c^Institute for Physics of Intelligence, School of Science, The University of Tokyo, Bunkyo-ku, Tokyo 113–0033, Japan; ^d^Department of Biomedical Engineering, The Pennsylvania State University, University Park, PA 16802; ^e^Department of Chemistry, The Pennsylvania State University, University Park, PA 16802; ^f^Department of Mathematics, The Pennsylvania State University, University Park, PA 16802

**Keywords:** active matter, active turbulence, bacteria, vortex, weakly nonlinear analysis

## Abstract

Active turbulence, or chaotic self-organized collective motion, is often observed in concentrated suspensions of motile bacteria and other systems of self-propelled interacting agents. To date, there is no fundamental understanding of how geometrical confinement orchestrates active turbulence and alters its physical properties. Here, by combining large-scale experiments, computer modeling, and analytical theory, we have identified a generic sequence of transitions occurring in bacterial suspensions confined in cylindrical wells of varying radii. With increasing the well’s radius, we observed that persistent vortex motion gives way to periodic vortex reversals, four-vortex pulsations, and then well-developed active turbulence. Using computational modeling and analytical theory, we have shown that vortex reversal results from the nonlinear interaction of the first three azimuthal modes that become unstable with the radius increase. The analytical results account for our key experimental findings. To further validate our approach, we reconstructed equations of motion from experimental data. Our findings shed light on the universal properties of confined bacterial active matter and can be applied to various biological and synthetic active systems.

## Significance

Biological and synthetic self-propelled entities, such as cultured cells, swimming bacteria, and active colloids, often exhibit complex collective motion. Controlling and rectifying such motion is crucial for developing microscopic active devices and sensors composed of swarming self-propelled particles. This study reveals how geometrical confinement transforms chaotic motion into a stabilized vortex and converts it into unsteady reversing configurations. The underlying mechanism is generic and thus applicable to various active systems. This work paves the way for design strategies for active devices grounded in robust theoretical insights.

Interacting self-propelled particles, often termed active matter, exhibit a remarkable tendency to self-organization and the onset of collective behavior. Being intrinsically out-of-equilibrium, active matter systems exhibit a slew of collective phenomena such as the spontaneous onset of long-range order (1–5), odd viscoelasticity (6), rectifications of chaotic flows (7–10), and reduction of the effective viscosity (11, 12). One of the most visible manifestations of collective dynamics in active matter systems is the emergence of self-sustained spatiotemporal chaotic flows termed active turbulence (13–18). In stark contrast to conventional Navier–Stokes turbulence, active turbulence, occurring for essentially zero Reynolds numbers, is characterized by the well-defined characteristic length scale. In the case of bacterial turbulence, this scale corresponds to typical vortex size, which is about 40 to 50 μm (14, 15). The existence of the typical vortex size allows transforming bacterial motion into stable vortex arrays under geometrical confinements (19–24) or in the presence of periodic obstacles (9, 10).

Experimental and computational studies of self-organization of bacterial and related active systems have shown that strong confinement, e.g., a cylindrical well, may suppress active turbulence and generate persistent vortex motion (19, 20, 22–24). However, a fundamental question on the nature of the transition from ordered states under strong confinement to chaotic motion in unconstrained systems remains open. Answering this question will shed light on intricate fundamental mechanisms of self-organization in a broad class of active systems under confinement.

In the context of active nematics exemplified by microtubules-motors assays, multiple experimental and numerical studies interrogated a transition from ordered quasi-stationary states to chaotic motion that occurs under the confinement in channels, rings, and wells (25–32). The primary observation is that the instability of static nematic configuration occurs via unbinding and subsequent chaotic motion of half-integer topological defects. Moreover, in the context of cytoplasmic streaming (31, 32), the onset of spontaneous circulation and consequent periodic modulations of the circulation rate were observed for the system confined in a cylindrical well. More specifically, the analysis predicted a supercritical instability of steady-state vortex with the increase in the well radius. The instability occurred via the gradual unbinding of two half-integer nematic defects with the amplitude of the oscillations vanishing at the critical radius.

However, it is unclear how these insights could be projected on polar systems, e.g. suspensions of swimming bacteria, where the polar symmetry of the systems would prohibit the above scenario. Also, in the bacterial suspensions, experimental investigations have been hindered by the difficulty in resolving the detailed dynamics very close to the transition point and the necessity of long-time measurements for evaluating the vortex stability.

Here, we examine the route to active turbulence by combining large-scale experiments, high-resolution numerical modeling, and analytical theory. We focused on a well-characterized active system: suspensions of swimming bacteria (5). We confined the suspensions into an array of isolated cylindrical wells comparable to the size of individual vortices. We systematically varied the wells’ radii to characterize the transition from stabilized vortices to bacterial turbulence. Increasing the well radius, we have detected reversals of vortex rotation as the first instability from a stable vortex. The reversals were also captured as periodic oscillations in our numerical simulations and analytical theory, unraveling a robust fundamental mechanism for the onset of polar active turbulence. In stark contrast to nematic systems (31, 32) where a local Hopf bifurcation results in small-amplitude periodic modulations of the steady-state circulation that keeps the same rotation direction (no reversals), in our case, the reversals occur via a global subcritical infinite period bifurcation with hysteresis, where the reversal period diverges at the threshold. Four-vortex pulsations follow the vortex reversal with a further increase in the radius. The observed transitions differ from the reversals caused by the viscoelasticity of the suspending fluid (33) or density gradients (34). Our analysis revealed that the reversal originate from the nonlinear interaction of the three lowest azimuthal modes near the linear instability threshold. To validate our theoretical arguments, we reconstructed equations of motion from experiential data. Our studies indicate that the vortex reversal is a generic precursor of turbulence-like behavior in bacterial and related active systems. Our findings provide insights into how geometrical confinement orchestrates spatiotemporal organization in a broad class of active systems.

## Results

### Experiment.

**Fig. 1.**
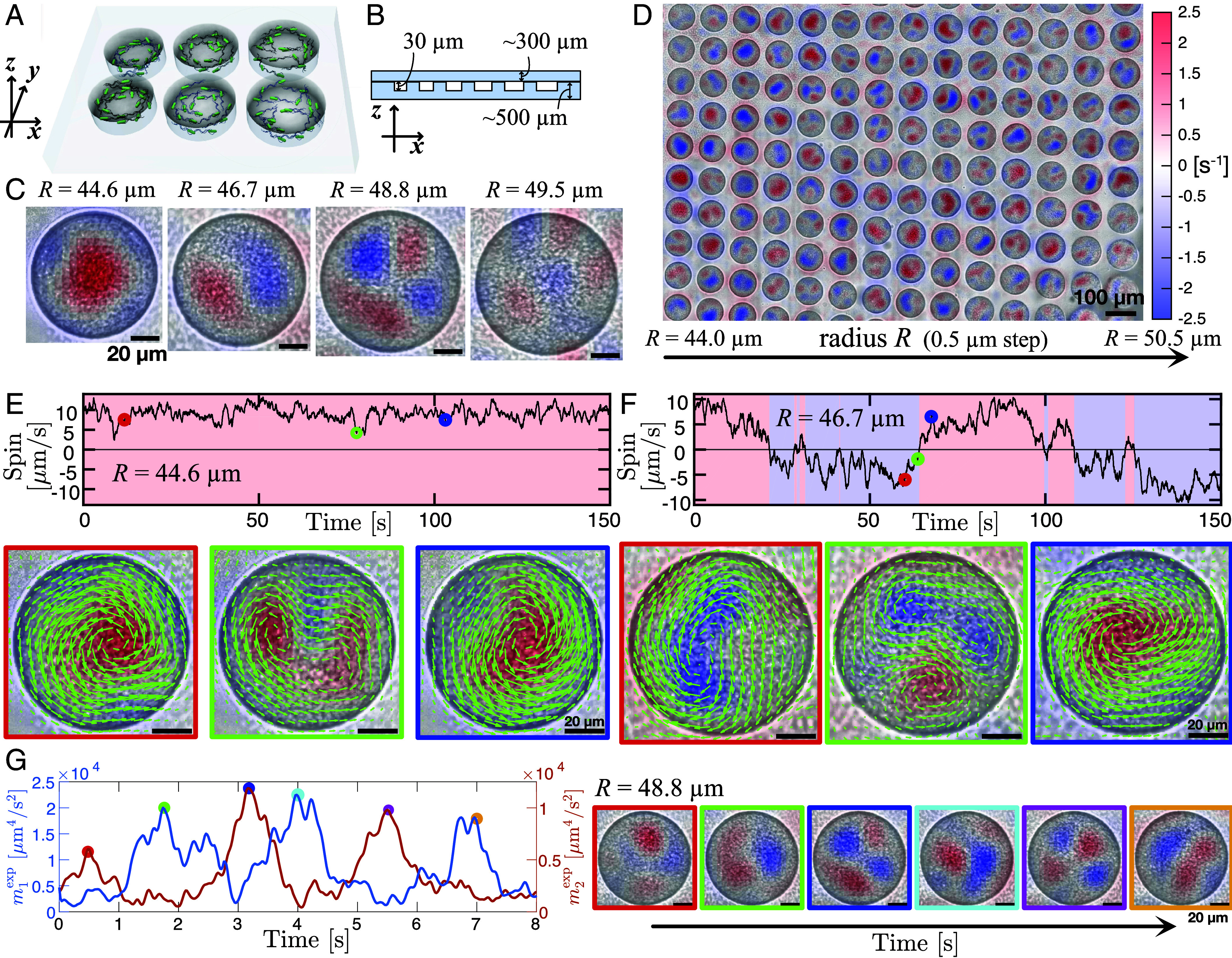
Transitions from a stabilized vortex to reversing vortices and a four-vortex state. 3D schematics (*A*) and side view (*B*) of the experimental setup. (*C*) Typical vorticity profiles of a single stabilized vortex (R=44.6 μm), reversing vortices (R=46.7 μm), a four-vortex state (R=48.8 μm), and a turbulent state (R=49.5 μm, *SI Appendix*, Fig. S5 and *Supplementary Note* 1G). Vorticity field ω is overlaid on the experimental snapshots. The color scales of the vorticity fields in all panels are identical and are indicated by the color bar in (*D*) (Movies S2–S5). (*D*) Experimental snapshot overlaid with the instantaneous vorticity field. Wells with the same radius are arranged vertically, with the radius increasing from *Left* to *Right*. All the 119 wells within this image out of ∼400 wells within the whole field of view (*SI Appendix*, Fig. S1 and Movie S1) were used for analysis; see *SI Appendix*, *Supplementary Note* 1E and Fig. S3 for the selection criteria. (*E* and *F*) Time series of spins for the wells with the radii of 44.6 μm (*E*) and 46.7 μm (*F*), respectively. The instantaneous velocity and vorticity fields are shown below the time series, with the colors of the rectangle corresponding to the time points highlighted by colored circles in the time series (Movies S2, S3, S6, and S7). (*G*) Antiphase relation of mode amplitudes $m_{1}^{\exp}$ and $m_{2}^{\exp}$ of the four-vortex state at R=48.8 μm. The instantaneous vorticity fields are shown on the right of the time series, with the colors of the rectangle corresponding to the time points highlighted by colored circles in the time series; see *SI Appendix*, *Supplementary Note* 4 and Movies S4 and S8.

**Fig. 2.**
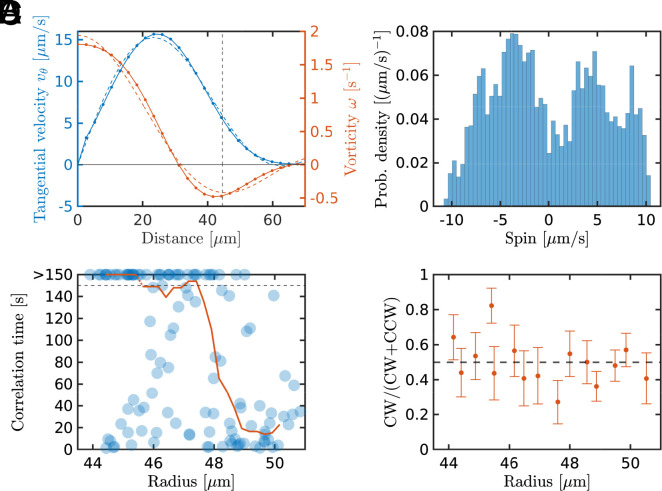
Characterization of vortex states. (*A*) Time-averaged velocity v (blue) and vorticity ω (red) profiles of a stabilized vortex shown in Fig. 1*E*. The vertical dashed line corresponds to the radius detected from the image analysis. Note that both velocity and vorticity penetrate beyond the well radius, due to the finite size of the PIV interrogation box and the application of Gaussian filtering; see *SI Appendix*, *Supplementary Note* 1I. The dashed lines are fits to the analytical solutions, Eq. **5**, for tangential velocity $v_{\theta }$ (blue) and vorticity ω (red) fields. (*B*) Spin probability density function for the reversing vortex state shown in Fig. 1*F*. (*C*) A scatter plot of the spin correlation time and the well radii detected from the image analysis. The red line represents the moving median of the scatter plot. The horizontal dashed line corresponds to the experimental duration, 150 s; see *SI Appendix*, *Supplementary Note* 1F for details. (*D*) Fraction of CW rotations as a function of the well radius. Error bars are the SEs.

We conducted experiments with suspensions of swimming bacteria confined in cylindrical wells, Fig. 1*A* and *B*. The height of the wells was set to 30 μm, which is smaller than the typical length scale of collective motion, ensuring effectively two-dimensional dynamics within each well. This constrained the range of radii for observation (*SI Appendix*, *Supplementary Note* 1G). Experiments were conducted simultaneously in an array of isolated wells of different radii (≈400 wells in total); see Fig. 1*D* and *SI Appendix*, Fig. S1, and Movies S1–S10. We observed stabilized vortices with steady rotational directions within the wells with small radii. For larger radii, the vortices exhibited a transition to unsteady configurations with reversing rotation directions. This observation is exemplified by the instantaneous vorticity field $\omega (r,t)=\hat{z}\cdot [\nabla \times v(r,t)]$ shown in Fig. 1*C*, where $\hat{z}$ is the unit vector in the z-direction. As one sees from Fig. 1*C*, the smaller wells hosted a single stabilized vortex with persistent rotation, with the velocity and vorticity profiles shown in Figs. 1*E* and 2*A*. In contrast to previous studies on bacterial suspensions confined in water-in-oil droplets (19, 20), we did not observe any counterrotating edge flows, suggesting different boundary conditions for collective motion.

To quantify the vortex rotation direction, we defined a spin variable for each well as,

[1] $$S_{i}\left(t\right):=\frac{\hat{z}\cdot \sum_{r\in i\text{-th well}}\left(r-r_{i}\right)\times v\left(r,t\right)}{\sum_{r\in i\text{-th well}}\left|r-r_{i}\right|},$$

where $r_{i}$ is the center of the i-th well, and the summations run over the area of the i-th well. As shown in Fig. 1 *E* and *F*, the spins for the small wells stayed almost constant and rarely flipped their signs over time, while the spins for the larger wells persistently alternated their signs, reflecting the reversals of vortices. The spin probability distribution for such a well with reversals exhibits a bimodal distribution, indicating the presence of two states with clockwise (CW, $S_{i}<0$) and counterclockwise (CCW, $S_{i}>0$) rotations (Fig. 2*B*); see *SI Appendix*, Fig. S6, *Supplementary Note* 1H, and Movie S10 for a well exhibiting faster reversals. Contrary to ref. (24), our setup uses two symmetric surfaces for the top and bottom to compensate for systematic bias in the rotation direction. Thus, the fraction of CW rotations as a function of the well’s radius was always ≈0.5, Fig. 2*D*. The absence of bias is crucial for characterizing the vortex reversals. The transition from a single stabilized vortex to reversing vortices was inspected through the spin correlation time, defined as the time at which the autocorrelation function of the spins decayed to 1/e; see *SI Appendix*, Fig. S4. The correlation time has successfully captured the transition at the radii of approximately 46 to 48 μm (Fig. 2*C*). For large wells’ radii, four-vortex pulsating states were observed as well, Fig. 1*C*. The pulsation was characterized by the kinetic energy of azimuthal modes corresponding to 2n vortices within a well (*SI Appendix*, *Supplementary Note* 4),

[2] $$m_{n}^{\exp}=\int_{0}^{R}rr{\left|\frac{1}{2\pi }\int \theta e^{-in\theta }v\left(r,\theta \right)\right|}^{2}.$$

By this mode analysis, we probed antiphase oscillation of the modes n=1 and n=2; see Fig. 1*G*.

Further increase in the radius destabilized the four-vortex pulsations, resulting in chaotic turbulent flow (Fig. 1*C* and *SI Appendix*, Fig. S5). For radii much smaller than those for the stabilized single-vortex state, such stable vortex formation was suppressed and we observed random-like motion of individual bacteria; see *SI Appendix*, Fig. S4 and Movie S9.

### Computational Modeling.

We performed numerical simulations using a phenomenological active fluid model, the Toner–Tu–Swift–Hohenberg equation (TTSHE) (5, 10, 15–17). The TTSHE qualitatively captures the bulk properties of polar active turbulence. It can describe the transformation of bacterial turbulence into stable vortex arrays in the presence of periodic obstacles (9, 10) and has been used to investigate the instability of the emergent order (35). In the vorticity representation, the dimensionless TTSHE is of the form (10):

[3] $$\begin{array}{c} \frac{\partial \omega }{\partial t}+\lambda v\cdot \nabla \omega =a\omega -b\nabla \times [|v{|}^{2}v]\\ \;-{\left(1+\nabla^{2}\right)}^{2}\omega -\gamma_{v}\nabla \times [K(r)v]-\gamma_{\omega }K(r)\omega , \end{array}$$

where λ, a, and b are constants, K(r)≥0 is a scalar field that dampens v and ω outside the well (K≃1) without affecting the inside (K≃0), and $\gamma_{v,\omega }>0$ are damping coefficients. In this dimensionless form, the vortex characteristic size is 2π. Following ref. 10, we adopt the parameter values $\left(\lambda ,a,b,\gamma_{v},\gamma_{\omega }\right)=\left(9,0.5,1.6,40,4\right)$ and impose three boundary conditions on well’s wall,

[4] v=0,ω=0atr=R.

Compared with the Navier–Stokes equation, the extra boundary condition ω=0 is imposed due to the higher-order differential operator ($\nabla^{4}$) in Eq. **3**. We solved Eq. **3** with the above boundary conditions in two dimensions by the pseudospectral method; see *Methods*.

**Fig. 3.**
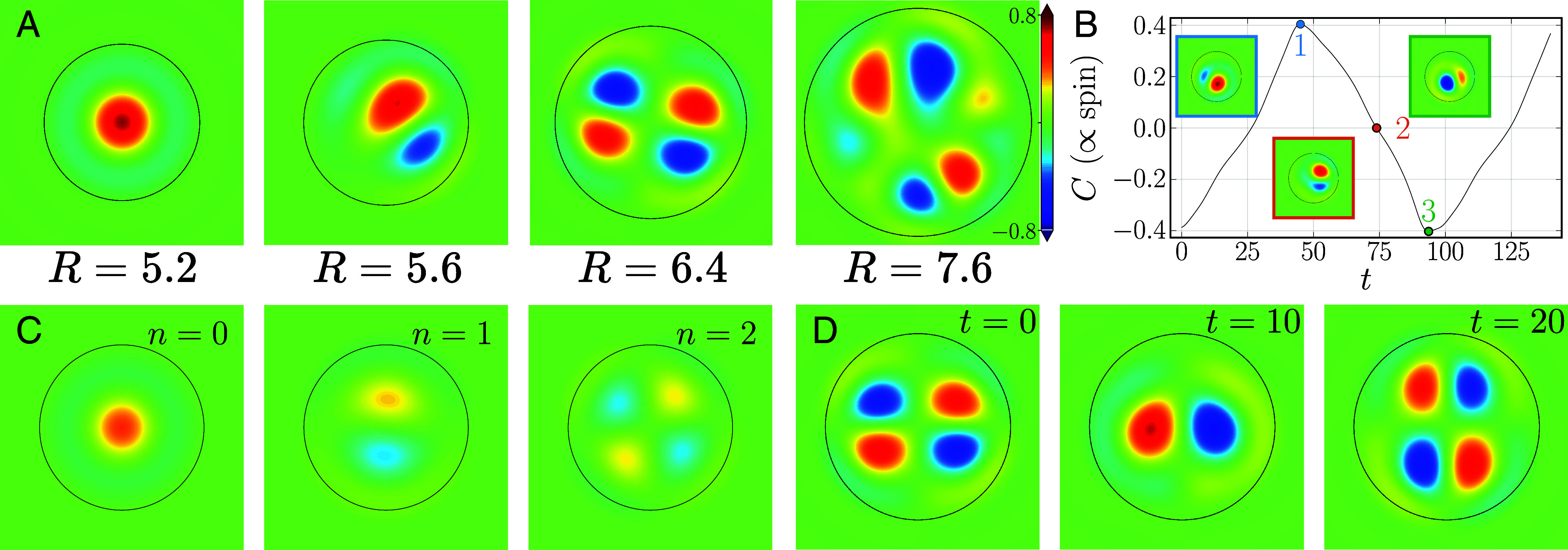
Computational modeling using the TTSHE. (*A*) Vorticity profiles obtained in the numerical simulations. Typical snapshots of a single stabilized vortex (R=5.2, Movie S11), periodically reversing two-vortex state (R=5.6, Movie S12), a pulsating four-vortex state (R=6.4, Movie S13), and a turbulent state (R=7.6, Movie S14) are shown. The color bar in all panels is the same. (*B*) Time series of the spin for the reversing two-vortex state (R=5.35, Movie S15). The instantaneous vorticity fields are shown as *Insets*, with the colors of the rectangle corresponding to the time points highlighted by colored circles in the time series. For computational convenience, instead of using the spin defined in (Eq. **1**), we plot the amplitude C of the zeroth azimuthal mode defined in (Eq. **6**), because C is proportional to the spin (*SI Appendix*, *Supplementary Note* 4). (*C*) Azimuthal mode decomposition of the instantaneous vorticity field for the reversing two-vortex state shown in the *Middle* panel of (*A*) (R=5.6, Movie S16), which is defined as ∫ω(r,θ)θ/2π for n=0 and $\int e^{-in\theta }\omega \left(r,\theta \right)\theta /2\pi +c.c.$ otherwise. (*D*) Snapshots of the pulsating four-vortex state (R=6.2, Movie S17).

Our simulations successfully reproduced the entire sequence of transitions observed in experiments, Fig. 3*A* and Movies S11–S17. We have found a single stable vortex for small radii. As the radius increases, the vortex becomes destabilized via infinite period bifurcation with hysteresis and yields a periodically reversing two-vortex state; see Fig. 3 *B* and *C*. It was demonstrated by the time series of the spin variable; see Fig. 3*B*. The infinite period bifurcation scenario (36) is consistent with the dependence of the correlation time in the experiment, Fig. 2*C*, which gradually decreases with the increase of the radius. Further increasing the radius, the reversing two-vortex state transforms into a pulsating four-vortex configuration, similarly to the experiment, Fig. 3*D*.

### Weakly Nonlinear Analysis.

**Fig. 4.**
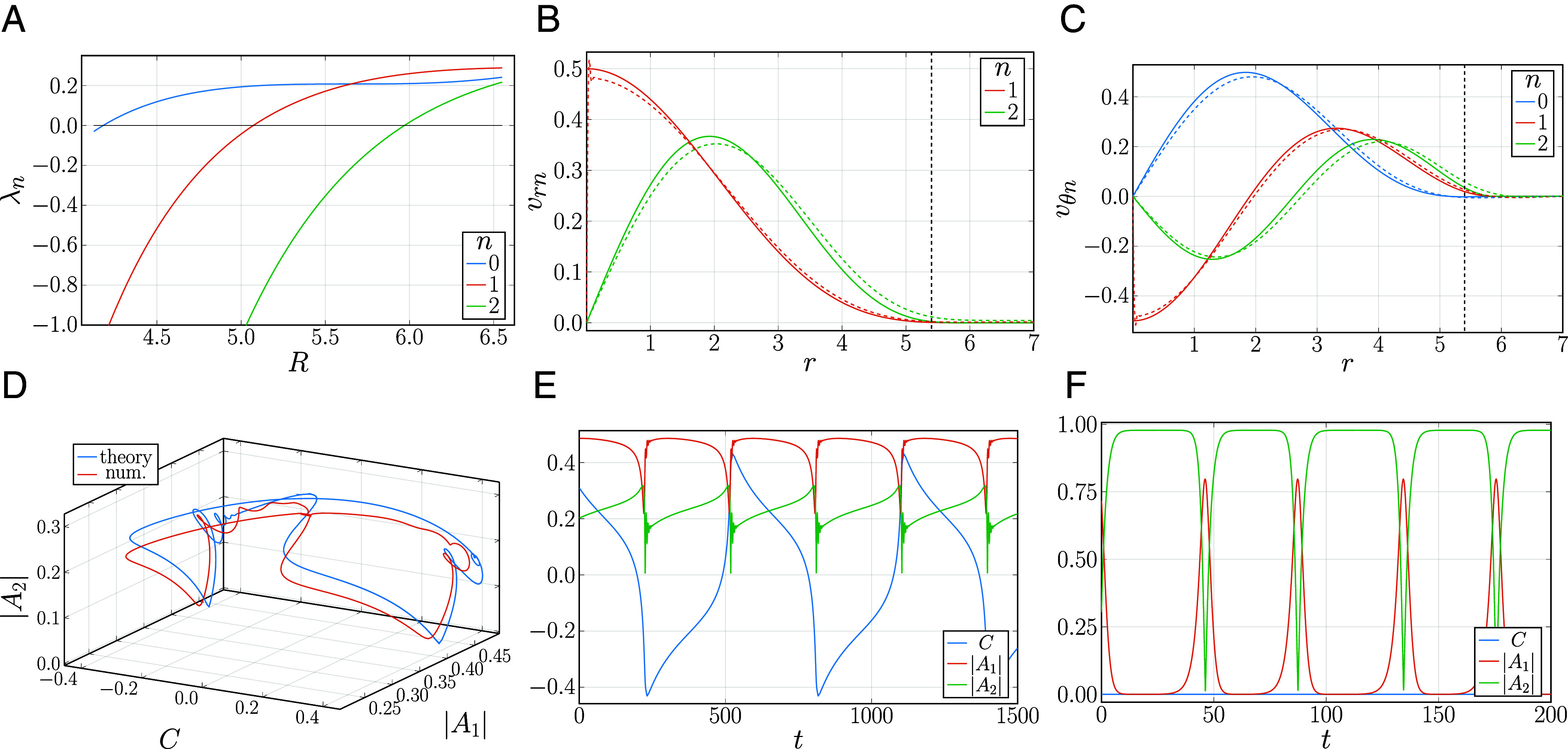
Analytical results. (*A*) Growth rates $\lambda_{n}$ vs. radius R for n=0,1,2. (*B* and *C*) Comparison of velocity profiles for the azimuthal modes with n=0,1,2 obtained from the linear theory (solid curves, R=5.9), Eq. **5**, and the TTSHE simulations (dashed curves, R=5.4). For the simulations, instantaneous profiles are plotted. The radius for the linear theory used in this comparison was roughly estimated by taking into account the leakage due to damping (*Methods* and *SI Appendix*, Fig. S11). The vertical black dashed line represents R=5.4 used for the simulations. (*D*) Comparison of trajectories in 3D phase space obtained by the solution of Eqs. **7**–**9** and the TTSHE. (*E* and *F*) Amplitudes $C,A_{1},A_{2}$ vs. time obtained from Eqs. **7**–**9** for R=5.9 (*E*) and R=7.0 (*F*); see Movies S18 and S19.

We examined the linear stability of Eq. **3** around v=0, yielding $\partial_{t}\omega =a\omega -{\left(1+\nabla^{2}\right)}^{2}\omega$. Its solution is of the form $\omega =\sum_{-\infty }^{\infty }\exp\left(\lambda_{n}t\right)\omega_{n}$,

[5] $$\omega_{n}=\left(G_{n+}J_{n}\left(k_{n+}r\right)+G_{n-}J_{n}\left(k_{n-}r\right)\right)\exp\left(in\theta \right),$$

where $\lambda_{n}$ are the growth rates of the corresponding azimuthal modes, $G_{n\pm }$ are constants, $J_{n}$ are the Bessel functions, $k_{n\pm }=\sqrt{1\pm \sqrt{a-\lambda_{n}}}$. Applying the boundary conditions to $\omega_{n}$ and solving the characteristic equation (*SI Appendix*, *Supplementary Note* 4), one finds the growth rates $\lambda_{n}$ vs. radius R. The results are shown in Fig. 4*A*. For small enough R, all $\lambda_{n}$ are negative, so that no vortex is excited, similarly to our experimental observation of noncoherent, random motion of individual bacteria for small wells. For R⪆4.2, $\lambda_{0}$ becomes positive, corresponding to the onset of the steady-state vortical motion observed in computational modeling and experiment; see *SI Appendix*, Fig. S11*B* for quantitative agreement between the numerical and analytical solutions. Then, with the gradual increase in R, higher rotational modes become unstable. We find that vortex reversal occurs at R≈5.88, when the first two modes (n=0,±1) are unstable, and the n=±2 mode is still stable but close to the threshold. This mode turns out to be excited due to nonlinear couplings with the modes n=0,±1, compare Fig. 4 *A* and *E* and see *SI Appendix*, *Supplementary Note* 4. The period of reversals diverges at the threshold radius, consistently with the infinite period bifurcation scenario; see *SI Appendix*, Fig. S10.

The radial vorticity and velocity profiles predicted by the linear analysis, Eq. **5**, are in excellent agreement with the numerical solutions of Eq. **3** without any fittings, Fig. 4 *B* and *C*. Furthermore, fitting the theoretical expression, Eq. **5**, for n=0 to experimental vorticity and velocity profiles of a stable vortex provides an excellent approximation as well; see Fig. 2*A* and *SI Appendix*, *Supplementary Note* 1I.

Next, we approximate the solution to Eq. **3** as a sum of the three lowest azimuthal modes with n=0,±1,±2,

[6] $$\omega =C\left(t\right)\omega_{0}\left(r\right)+\left[A_{1}\left(t\right)e^{i\theta }\omega_{1}\left(r\right)+A_{2}\left(t\right)e^{2i\theta }\omega_{2}\left(r\right)+\;\text{c.c.}\right]$$

Here, $\omega_{0},\omega_{1},\omega_{2}$ are the eigenfunctions obtained from linear stability analysis. For definiteness, the eigenmodes are normalized by their kinetic energy; see *SI Appendix*, *Supplementary Note* 4 and Eqs. **S35** and **S36**. $C\left(t\right),A_{1}\left(t\right),A_{2}\left(t\right)$ are slowly varying amplitudes that are derived from the corresponding orthogonality leading to a set of normal form Eqs. **7**–**9**; see *Methods*.

Eqs. **7**–**9** faithfully reproduce the numerical results from Eq. **3** without further approximation; see Fig. 4. Specifically, for small radii, Eqs. **7**–**9** reproduce a stable vortex solution as shown in Fig. 3*A*. Then, with the increase in R, the infinite period bifurcation to a limit cycle is faithfully captured. Furthermore, even the details of the time dependence of each azimuthal mode closely agree with those of the numerical solutions of Eq. **3**; see Fig. 4*D* and *SI Appendix*, Fig. S11 *C* and *D*. In the reversing vortex state, displayed in Fig. 4*E*, all three amplitudes $C,A_{1},A_{2}$ are nonzero; see Movie S18. With the further increase in R, a transition from a reversing state to a pulsating four-vortex solution occurs; see Fig. 4*F* and Movie S19. Here, the zero mode, n=0, is suppressed, and the first and second modes $A_{1},A_{2}$ pulsate in antiphase. As shown in Fig. 1*G*, this antiphase relation was indeed observed experimentally, further demonstrating the quantitative agreements among the experimental, numerical, and analytical results. The normal form analysis indicates that the transition to vortex reversals and other time-dependent states is a result of resonant nonlinear interaction among the three lowest azimuthal modes. This behavior only exists for sufficiently large values of the nonlinear advection term λv·∇ω in Eq. **3**, which controls the resonant three-mode interaction. No limit cycles were found for λ⪅3.75.

### Validating Equations of Motion.

The use of the TTSHE was validated through regression analysis of our experimental data; see *SI Appendix*, *Supplementary Note* 2 for details. Similar approaches were used in refs. 37 and 38. In addition to the TTSHE, we tested another model for bacterial turbulence, the Nikolaevskiy equation, which includes $\nabla^{6}v$ term but no cubic nonlinearity ${\left|v\right|}^{2}v$ nor linear term v (39–41). The TTSHE outperformed the Nikolaevskiy equation in terms of the residuals, justifying our numerical and theoretical approaches; see *SI Appendix*, Figs. S7–S9 and Tables S1–S3. The regression for the two-vortex reversing state shown in Fig. 1*F* yields $\lambda_{\dim}=1.69\pm 0.38$ for the dimensional TTSHE, proving the presence of the advection term with λ>1, larger than λ=1 for the Naiver-Stokes equation. Transforming the TTSHE into the form of Eq. **3** with characteristic values in the unconstrained bacterial turbulence (velocity V≈50 μm/s, length scale L≈40 μm, and time scale T≈0.5 s) yields $\lambda_{\mathrm{nondim}}=\frac{\mathrm{VT}}{L}\lambda_{\dim}\approx 4.2$ in the dimensionless TTSHE. It is consistent with our theoretical prediction of λ⪆3.75 for the onset of oscillations.

## Concluding Remarks

We observed a generic route to active turbulence in confined suspensions of swimming bacteria: a single steady vortex gives way to a reversing vortex pair, four pulsating vortices, and then to a well-developed spatiotemporal chaos. The fact that the entire bifurcation sequence is reproduced by a generic phenomenological model for active turbulence reveals the universal fundamental mechanism governing the transition: resonant interaction of the three lowest azimuthal modes associated with cylindrical confinement. Furthermore, the onset of the periodic reversal relies on the finite value of the Navier–Stokes-like advection term in the phenomenological model of active turbulence (10, 15, 17, 42). The regression of experimental data also reliably corroborates the presence of the advection term with its coefficient λ>1 in the effective equation. These findings suggest that the observed transitions should also occur in a broad class of active self-propelled systems under confinement. This robust mechanism is presumably responsible for the onset of reversing edge currents numerically observed in ref. 43 and is not sensitive to the details of boundary conditions or geometry (42). Furthermore, the observed transitions occur in a Newtonian fluid environment with homogeneous activity and density. Viscoelasticity or anisotropy may only affect the details of the transitions (33, 44, 45). The oxygen supply through the top and bottom polydimethylsiloxane (PDMS) membranes realized the homogeneity of the system, excluding the previously proposed reversal mechanism driven by density gradients arising from nonuniform oxygen supply limited to the circumference (34). This generic mechanism is based on the three-mode resonant interaction and should be relevant for the variety of biological and synthetic active systems, e.g., Janus colloids (3, 4, 46).

Another intriguing aspect is the effect of chirality. Since bacteria are chiral objects due to counterrotation of the body and helical flagella (47–50), there could be an asymmetry between CW/CCW rotating vortices (24). In this work, a sustained effort was undertaken to make the upper/bottom surfaces of the wells as identical as possible to suppress the asymmetry. While a minor chiral shift does not affect the transition sequence, it could introduce slightly different thresholds for the onsets of vortex oscillations of opposite chirality.

The current experiment is unavoidably susceptible to strong fluctuations discarded in the theoretical description. For example, the number of bacteria within a single microscopic well is about ∼10^4^ bacteria/well. The dynamics of such a small bacterial population is intrinsically stochastic. Therefore, understanding how noise affects the nature of transitions and exploring ways to tame and control the fluctuating active dynamics would be of interest to future studies.

Finally, the controls and rectification of vortices in confined active matter open up possibilities for engineering out-of-equilibrium systems. For instance, weak coupling between neighboring wells may realize a “bacterial lattice clock,” in which reversing vortex pairs synchronize and exhibit higher regularity and persistence. The reversing or pulsating vortices may be useful for mixing at low Reynolds numbers. Taming the fluctuations in active systems based on the fundamental instability uncovered in this work provides design principles for functioning active devices, such as biosensors or microrobotic swarms for targeted drug delivery, precision surgery, or detoxification (51, 52).

## Methods

## Materials and Methods

### Experimental Details.

Bacteria *Bacillus subtilis* (strain: 1085) were grown in Terrific Broth (T9179, Sigma-Aldrich) growth medium until optical density (OD) achieved $OD_{600\;nm}\approx$1. After concentrating the suspension 180-fold, it was sandwiched between two thin PDMS membranes to facilitate sufficient oxygen supply for sustaining high bacterial motility. The bottom PDMS membrane was patterned with 30-μm-deep multiple microscopic wells with the radius ranging from 44 to 51 μm with 0.5 μm increments (Fig. 1*A*, *B*, and *D* and *SI Appendix*, Figs. S1 and S2). To overcome systematic errors arising from different preparations of bacterial cultures and slight density variations caused during the confinement process, we simultaneously observed ∼400 wells (19 radii, ∼20 wells for each radius) in a single field of view by using an inverted microscope equipped with a large-sensor sCMOS camera (Kinetix, Teledyne Photometrics, 3,200 × 3,200 pixels) and a 10× objective lens, realizing the 2.1 mm × 2.1 mm field of view (*SI Appendix*, Fig. S1). It allowed resolving the bacterial dynamics very close to the transition point. We captured the movies at 50 fps for 150 s and analyzed the bacterial velocity fields $v\left(r,t\right)=\left(v_{x},v_{y}\right)$ using the particle image velocimetry (PIV). The duration was limited to 150 s to ensure statistical stationarity, which is eventually spoiled by the gradual decrease in bacterial activity. See *SI Appendix*, *Supplementary Note* 1A–D for the detailed protocols.

### Computational Details.

Eq. **3** was solved by the pseudospectral method in a two-dimensional periodic 40.96×40.96 domain, discretized as the 8,192 × 8,192 square lattice. Spatial derivatives were handled by the fast Fourier transform; see *SI Appendix*, *Supplementary Note* 3. Time update was performed in the Fourier space, with the time step Δt=0.01. To accelerate simulations, we performed the whole computation on GPUs (NVIDIA RTX A6000 or A100).

The damping wall implemented in Eq. **3** with the kernel K(r) permits some leakage outside of the well radius R. We calibrated R to account for the leakage and defined the effective radius $R_{\mathrm{eff}}$, where the velocity and vorticity vanish. $R_{\mathrm{eff}}$ is calculated as the root of $\int v_{\theta }\left(r,\theta \right)\theta$ (the zeroth azimuthal mode); see *SI Appendix*, Fig. S11. We obtained $R_{\mathrm{eff}}-R\approx 0.5$. The zeroth mode amplitude C in (Eq. **6**) provides a convenient measure of the spin ((Eq. **1**)) up to a certain prefactor. For the details of the numerical mode decomposition and related quantities, see *SI Appendix*, *Supplementary Note* 4.

### Normal form Equations.

Substituting Eq. **6** into Eq. **3**, and implementing the orthogonality conditions, we obtain the set of equations for amplitudes $C,A_{1},A_{2}$,

[7] $$\begin{array}{cc} \partial_{t}C & =\lambda_{0}C-c_{1}C^{3}-c_{2}C|A_{1}{|}^{2}-c_{3}C{\left|A_{2}\right|}^{2}\\ & \;-2c_{4}\;\text{Re}A_{2}{A_{1}^{*}}^{2}, \end{array}$$

[8] $$\begin{array}{cc} \partial_{t}A_{1} & =\lambda_{1}A_{1}-b_{1}A_{1}|A_{1}{|}^{2}-b_{2}A_{1}C^{2}-b_{3}A_{1}{\left|A_{2}\right|}^{2}\\ & \;-b_{4}CA_{2}A_{1}^{*}+\delta_{1}A_{1}C+\gamma_{1}A_{2}A_{1}^{*}, \end{array}$$

[9] $$\begin{array}{cc} \partial_{t}A_{2} & =\lambda_{2}A_{2}-a_{1}A_{2}|A_{2}{|}^{2}-a_{2}A_{2}C^{2}-a_{3}A_{2}{\left|A_{1}\right|}^{2}\\ & \;-a_{4}CA_{1}^{2}+\delta_{2}A_{2}C+\gamma_{2}A_{1}^{2}, \end{array}$$

where $\lambda_{0,1,2}$ are the linear growth rates; other coefficients are integrals over the nonlinearities, *SI Appendix*, *Supplementary Note* 4. All coefficients are calculated using a Mathematica script provided as a *SI Appendix*.

## Supplementary Material

Appendix 01 (PDF)

Movie S1. Movie of the whole field of view (2.1 mm × 2.1 mm), corresponding to Fig. S1. The initial 20 seconds of the data are presented at twice the real speed (2×). The spatial resolution is lowered to reduce the file size.

Movie S2. The vorticity and velocity fields of the single stabilized vortex (*R* = 44.6 *μ*m). The color bar of the vorticity field is the same as in Fig. 1. The movie is played at real-time speed. The scale bar represents 20 *μ*m.

Movie S3. The vorticity and velocity fields of the reversing vortices (*R* = 46.7 *μ*m). The color bar of the vorticity field is the same as in Fig. 1. The movie is played at real-time speed. The scale bar represents 20 *μ*m.

Movie S4. The vorticity and velocity fields of the four-vortex state (*R* = 48.8 *μ*m). The color bar of the vorticity field is the same as in Fig. 1. The movie is played at real-time speed. The scale bar represents 20 *μ*m.

Movie S5. The vorticity and velocity fields of the turbulent state (*R* = 49.5 *μ*m). The color bar of the vorticity field is the same as in Fig. 1. The movie is played at real-time speed. The scale bar represents 20 *μ*m.

Movie S6. The velocity field of the single stabilized vortex (*R* = 44.6 *μ*m). The velocity vectors are colored based on the sign of the local vorticity: yellow for positive and green for negative. The movie is played at 4× real-time speed. The scale bar represents 20 *μ*m.

Movie S7. The velocity field of the reversing vortices (*R* = 46.7 *μ*m). The velocity vectors are colored based on the sign of the local vorticity: yellow for positive and green for negative. The movie is played at 4× real-time speed. The scale bar represents 20 *μ*m.

Movie S8. The velocity field of the four-vortex state (*R* = 48.8 *μ*m). The velocity vectors are colored based on the sign of the local vorticity: yellow for positive and green for negative. The movie is played at 4× real-time speed. The scale bar represents 20 *μ*m.

Movie S9. The movie of the non-coherent random motion in the small well (*R* = 42.9 *μ*m). The movie is played at real-time speed. The scale bar represents 20 *μ*m.

Movie S10. The vorticity and velocity fields of the reversing vortices (*R* = 48.0 *μ*m). The color bar of the vorticity field is the same as in Fig. 1. The movie is played at real-time speed. The scale bar represents 20 *μ*m.

Movie S11. Vorticity field of the single stabilized vortex (Fig. 3(a), left), numerically computed at *R* = 5.2.

Movie S12. Vorticity field of the reversing two-vortex state (Fig. 3(a), the second from the left), numerically computed at *R* = 5.6.

Movie S13. Vorticity field of the pulsating four-vortex state (Fig. 3(a), the third from the left), numerically computed at *R* = 6.4.

Movie S14. Vorticity field of the turbulent state (Fig. 3(a), right), numerically computed at *R* = 7.6.

Movie S15. Vorticity field of the reversing two-vortex state(Fig. 3(b)), numerically computed at *R* = 5.35.

Movie S16. Azimuthal Fourier decomposition of the vorticity field of the reversing two-vortex state at *R* = 5.6 (Fig. 3(c)). The original vorticity field and the modes n = 0, 1, 2 are shown.

Movie S17. Vorticity field of the pulsating four-vortex state (Fig. 3(d)), numerically computed at *R* = 6.2.

Movie S18. Vorticity field and its azimuthal Fourier decomposition of the analytically calculated reversing two-vortex state at *R* = 5.9 (Fig. 4(e)). The definitions of the modes are the same as in Supplementary Movie 12 and Fig. 3(c).

Movie S19. Vorticity field and its azimuthal Fourier decomposition of the analytically calculated pulsating four-vortex state at *R* = 7.0 (Fig. 4(f)). The definitions of the modes are the same as in Supplementary Movie 12 and Fig. 3(c).

Movie S20.

## Data Availability

All the relevant experimental and numerical data, the MATLAB codes for analyzing the experimental data, Python scripts for the numerical simulations of the TTSHE, and the Mathematica code for analytical theory are deposited on Zenodo (53). Although only the initial parts of the experimental movies were deposited due to the size limit of the repository, the complete image sequences can be obtained by contacting the authors at nishiguchi@phys.isct.ac.jp. The Mathematica code for analytical theory is also included in *SI Appendix*.

## References

[1] D. Nishiguchi, K. H. Nagai, H. Chaté, M. Sano, Long-range nematic order and anomalous fluctuations in suspensions of swimming filamentous bacteria. Phys. Rev. E **95**, 020601(R) (2017). 28297912 10.1103/PhysRevE.95.020601

[2] H. Chaté, Dry aligning dilute active matter. Annu. Rev. Condens. Matter Phys. **11**, 189–212 (2020).

[3] J. Iwasawa, D. Nishiguchi, M. Sano, Algebraic correlations and anomalous fluctuations in ordered flocks of Janus particles fueled by an AC electric field. Phys. Rev. Res. **3**, 043104 (2021).

[4] D. Nishiguchi, Deciphering long-range order in active matter: Insights from swimming bacteria in quasi-2D and electrokinetic janus particles. J. Phys. Soc. Jpn. **92**, 121007 (2023).

[5] I. S. Aranson, Bacterial active matter. Rep. Prog. Phys. **85**, 076601 (2022). 10.1088/1361-6633/ac723d 35605446

[6] M. Fruchart, C. Scheibner, V. Vitelli, Odd viscosity and odd elasticity. Annu. Rev. Condens. Matter Phys. **14**, 471–510 (2023).

[7] A. Sokolov, M. M. Apodaca, B. A. Grzybowski, I. S. Aranson, Swimming bacteria power microscopic gears. Proc. Natl. Acad. Sci. U.S.A. **107**, 969–974 (2010). 20080560 10.1073/pnas.0913015107 PMC2824308

[8] C. O. Reichhardt, C. Reichhardt, Ratchet effects in active matter systems. Annu. Rev. Condens. Matter Phys. **8**, 51–75 (2017).

[9] D. Nishiguchi, I. S. Aranson, A. Snezhko, A. Sokolov, Engineering bacterial vortex lattice via direct laser lithography. Nat. Commun. **9**, 4486 (2018). 30367049 10.1038/s41467-018-06842-6 PMC6203773

[10] H. Reinken , Organizing bacterial vortex lattices by periodic obstacle arrays. Commun. Phys. **3**, 76 (2020).

[11] A. Sokolov, I. S. Aranson, Reduction of viscosity in suspension of swimming bacteria. Phys. Rev. Lett. **103**, 148101 (2009). 19905604 10.1103/PhysRevLett.103.148101

[12] V. A. Martinez , A combined rheometry and imaging study of viscosity reduction in bacterial suspensions. Proc. Natl. Acad. Sci. U.S.A. **117**, 2326–2331 (2020). 31964833 10.1073/pnas.1912690117 PMC7007524

[13] A. Sokolov, I. S. Aranson, J. O. Kessler, R. E. Goldstein, Concentration dependence of the collective dynamics of swimming bacteria. Phys. Rev. Lett. **98**, 158102 (2007). 17501387 10.1103/PhysRevLett.98.158102

[14] A. Sokolov, I. S. Aranson, Physical properties of collective motion in suspensions of bacteria. Phys. Rev. Lett. **109**, 248109 (2012). 23368392 10.1103/PhysRevLett.109.248109

[15] H. H. Wensink , Meso-scale turbulence in living fluids. Proc. Natl. Acad. Sci. U.S.A. **109**, 14308–14313 (2012). 22908244 10.1073/pnas.1202032109 PMC3437854

[16] J. Dunkel , Fluid dynamics of bacterial turbulence. Phys. Rev. Lett. **110**, 228102 (2013). 23767750 10.1103/PhysRevLett.110.228102

[17] J. Dunkel, S. Heidenreich, M. Bär, R. E. Goldstein, Minimal continuum theories of structure formation in dense active fluids. New J. Phys. **15**, 045016 (2013).

[18] K. Qi, E. Westphal, G. Gompper, R. G. Winkler, Emergence of active turbulence in microswimmer suspensions due to active hydrodynamic stress and volume exclusion. Commun. Phys. **5**, 49 (2022).

[19] H. Wioland, F. G. Woodhouse, J. Dunkel, J. O. Kessler, R. E. Goldstein, Confinement stabilizes a bacterial suspension into a spiral vortex. Phys. Rev. Lett. **110**, 268102 (2013). 23848925 10.1103/PhysRevLett.110.268102

[20] E. Lushi, H. Wioland, R. E. Goldstein, Fluid flows created by swimming bacteria drive self-organization in confined suspensions. Proc. Natl. Acad. Sci. U.S.A. **111**, 9733–9738 (2014). 24958878 10.1073/pnas.1405698111 PMC4103334

[21] H. Wioland, E. Lushi, R. E. Goldstein, Directed collective motion of bacteria under channel confinement. New J. Phys. **18**, 075002 (2016).

[22] H. Wioland, F. G. Woodhouse, J. Dunkel, R. E. Goldstein, Ferromagnetic and antiferromagnetic order in bacterial vortex lattices. Nat. Phys. **12**, 341–345 (2016). 27213004 10.1038/nphys3607 PMC4869837

[23] K. Beppu , Geometry-driven collective ordering of bacterial vortices. Soft Matter **13**, 5038–5043 (2017). 28702666 10.1039/c7sm00999b

[24] K. Beppu , Edge current and pairing order transition in chiral bacterial vortices. Proc. Natl. Acad. Sci. U.S.A. **118**, e2107461118 (2021). 34561308 10.1073/pnas.2107461118 PMC8488682

[25] K. T. Wu , Transition from turbulent to coherent flows in confined three-dimensional active fluids. Science **355**, 1284 (2017). 10.1126/science.aal1979 28336609

[26] A. Opathalage , Self-organized dynamics and the transition to turbulence of confined active nematics. Proc. Natl. Acad. Sci. U.S.A. **116**, 4788–4797 (2019). 30804207 10.1073/pnas.1816733116 PMC6421422

[27] J. Hardoüin , Reconfigurable flows and defect landscape of confined active nematics. Commun. Phys. **2**, 121 (2019).

[28] J. Hardoüin , Active boundary layers in confined active nematics. Nat. Commun. **13**, 6675 (2022). 36335213 10.1038/s41467-022-34336-z PMC9637202

[29] C. D. Schimming, C. Reichhardt, C. Reichhardt, Friction-mediated phase transition in confined active nematics. Phys. Rev. E **108**, L012602 (2023). 37583137 10.1103/PhysRevE.108.L012602

[30] M. Ravnik, J. M. Yeomans, Confined active nematic flow in cylindrical capillaries. Phys. Rev. Lett. **110**, 026001 (2013). 23383919 10.1103/PhysRevLett.110.026001

[31] F. G. Woodhouse, R. E. Goldstein, Spontaneous circulation of confined active suspensions. Phys. Rev. Lett. **109**, 168105 (2012). 23215137 10.1103/PhysRevLett.109.168105

[32] F. G. Woodhouse, R. E. Goldstein, Cytoplasmic streaming in plant cells emerges naturally by microfilament self-organization. Proc. Natl. Acad. Sci. U.S.A. **110**, 14132–14137 (2013). 23940314 10.1073/pnas.1302736110 PMC3761564

[33] S. Liu, S. Shankar, M. C. Marchetti, Y. Wu, Viscoelastic control of spatiotemporal order in bacterial active matter. Nature **590**, 80–84 (2021). 33536650 10.1038/s41586-020-03168-6

[34] A. E. Hamby, D. K. Vig, S. Gaines, C. W. Wolgemuth, Swimming bacteria power microspin cycles. Sci. Adv. **4**, eaau0125 (2018). 30585288 10.1126/sciadv.aau0125 PMC6300399

[35] H. Reinken, S. Heidenreich, M. Bär, S. H. L. Klapp, Pattern selection and the route to turbulence in incompressible polar active fluids. New J. Phys. **26**, 063026 (2024).

[36] S. H. Strogatz, Ed., “Nonlinear dynamics and chaos: With applications to physics, biology, chemistry, and engineering (studies in nonlinearity)” in Nonlinear Dynamics and Chaos: With Applications to Physics, Biology, Chemistry, and Engineering (Studies in Nonlinearity) (CRC Press, 2001), pp. 262–264.

[37] S. L. Brunton, J. L. Proctor, J. N. Kutz, Discovering governing equations from data by sparse identification of nonlinear dynamical systems. Proc. Natl. Acad. Sci. U.S.A. **113**, 3932–3937 (2016). 27035946 10.1073/pnas.1517384113 PMC4839439

[38] R. Supekar , Learning hydrodynamic equations for active matter from particle simulations and experiments. Proc. Natl. Acad. Sci. U.S.A. **120**, e2206994120 (2023). 36763535 10.1073/pnas.2206994120 PMC9963139

[39] I. A. Beresnev, V. N. Nikolaevskiy, A model for nonlinear seismic waves in a medium with instability. Phys. D Nonlinear Phenom. **66**, 1–6 (1993).

[40] M. I. Tribelsky, K. Tsuboi, New scenario for transition to turbulence? Phys. Rev. Lett. **76**, 1631 (1996). 10060478 10.1103/PhysRevLett.76.1631

[41] J. Słomka, J. Dunkel, Spontaneous mirror-symmetry breaking induces inverse energy cascade in 3D active fluids. Proc. Natl. Acad. Sci. U.S.A. **114**, 2119–2124 (2017). 28193853 10.1073/pnas.1614721114 PMC5338532

[42] S. Shiratani, K. A. Takeuchi, D. Nishiguchi, Route to turbulence via oscillatory states in polar active fluid under confinement. arXiv [Preprint] (2023). http://arxiv.org/abs/2304.03306 (Accessed 28 July 2024).

[43] H. Matsukiyo, J.-I. Fukuda, Oscillating edge current in polar active fluid. Phys. Rev. E **109**, 054604 (2024). 38907507 10.1103/PhysRevE.109.054604

[44] W. Liao, I. S. Aranson, Viscoelasticity enhances collective motion of bacteria. PNAS Nexus **2**, pgad291 (2023). 37719751 10.1093/pnasnexus/pgad291 PMC10503537

[45] S. Zhou, A. Sokolov, O. D. Lavrentovich, I. S. Aranson, Living liquid crystals. Proc. Natl. Acad. Sci. U.S.A. **111**, 1265 (2014). 24474746 10.1073/pnas.1321926111 PMC3910648

[46] D. Nishiguchi, M. Sano, Mesoscopic turbulence and local order in Janus particles self-propelling under an ac electric field. Phys. Rev. E **92**, 052309 (2015). 10.1103/PhysRevE.92.052309 26651697

[47] W. R. DiLuzio , Escherichia coli swim on the right-hand side. Nature **435**, 1271–1274 (2005). 15988531 10.1038/nature03660

[48] Marcos, H. C. Fu, T. R. Powers, R. Stocker, Bacterial rheotaxis. Proc. Natl. Acad. Sci. U.S.A. **109**, 4780–4785 (2012). 22411815 10.1073/pnas.1120955109 PMC3324032

[49] G. Jing, A. Zöttl, É. Clément, A. Lindner, Chirality-induced bacterial rheotaxis in bulk shear flows. Sci. Adv. **6**, eabb2012 (2020). 32695880 10.1126/sciadv.abb2012 PMC7351478

[50] D. Grober , Unconventional colloidal aggregation in chiral bacterial baths. Nat. Phys. **19**, 1680–1688 (2023).

[51] J. Li, B. Esteban-Fernández de Ávila, W. Gao, L. Zhang, J. Wang, Micro/nanorobots for biomedicine: Delivery, surgery, sensing, and detoxification. Sci. Robot. **2**, eaam6431 (2017). 31552379 10.1126/scirobotics.aam6431 PMC6759331

[52] J. Li, M. Pumera, 3d printing of functional microrobots. Chem. Soc. Rev. **50**, 2794–2838 (2021). 33470252 10.1039/d0cs01062f

[53] D. Nishiguchi, S. Shiratani, I. S. Aranson, K. A. Takeuchi, Codes and data used in “vortex reversal is a precursor of confined bacterial turbulence”. Zenodo. 10.5281/zenodo.14553935. Accessed 25 December 2024. PMC11929451 40085657

