# Supplementary material for: Vortex reversal is a precursor of confined bacterial turbulence
Source: Proc Natl Acad Sci U S A. 2025 Mar 14;122(11):e2414446122. doi: 10.1073/pnas.2414446122 (PMC11929451; doi:10.1073/pnas.2414446122)
Supplement: Supplementary file 1 — Appendix 01 (PDF) [file pnas.2414446122.sapp.pdf]

Supplementary Material for
"Vortex reversal is a precursor of confined bacterial turbulence"

Daiki Nishiguchi,[1,2,*] Sora Shiratani,[2] Kazumasa A. Takeuchi,[2,3] and Igor S. Aranson[4,2]

[1]*Department of Physics, Institute of Science Tokyo, 2–12–1 Ookayama, Meguro-ku, Tokyo 152–8551, Japan*
[2]*Department of Physics, The University of Tokyo, 7–3–1 Hongo, Bunkyo-ku, Tokyo 113–0033, Japan*
[3]*Institute for Physics of Intelligence, The University of Tokyo, 7–3–1 Hongo, Bunkyo-ku, Tokyo 113–0033, Japan*
[4]*Departments of Biomedical Engineering, Chemistry, and Mathematics, The Pennsylvania State University, University Park, Pennsylvania 16802, USA*
(Dated: February 11, 2025)

I. SUPPLEMENTARY NOTE 1: EXPERIMENTAL DETAILS

A. Bacterial culture

Bacteria *Bacillus subtilis* (strain 1085) were first grown on an LB (Luria-Bertani) agar plate (BD Difco LB Broth Miller + 1.5 w% agar) at 37°C. The grown bacterial colony was transferred into 13 mL of Terrific Broth (T9179, Sigma-Aldrich) medium in a glass test tube, sealed with a cap, and cultured at 37°C at 200 rpm. When it reached $OD_{600\ \mathrm{nm}}$ ≈0.8–1.0, the test tube was taken out from the incubator and placed at room temperature (23°C) for 45 minutes so that the bacteria became adjusted to the temperature before the experiments. The bacterial suspension was centrifuged at 3000 rpm (1057 rcf) for 2 minutes and concentrated 180-fold by removing the supernatant. The concentrated bacterial suspension was confined into the PDMS device for observation.

B. Microfabrication

The fabrication of the microfluidic device was performed with the standard soft lithography. The photoresist SU-8 3050 was spread on a silicon wafer at the thickness of 30 μm, and circular patterns with multiple diameters were drawn with a maskless aligner μMLA (Heidelberg Instruments) and developed following the standard protocol. Then, dimethylpolysiloxane (PDMS, Sylgard 184, Dow) was poured onto the patterned silicon wafer to form the layer of the thickness $\sim$ 500 μm. We also poured PDMS onto another non-patterned bare silicon wafer to make a $\sim$ 300-μm-thick flat PDMS membrane to be used as the top plate. The cured PDMS membranes were peeled off from the silicon wafers. The plasma cleaning was applied to the membranes to make the surface hydrophilic so that the bacterial suspensions can smoothly enter the small circular wells.

We placed the concentrated bacterial suspension on the patterned PDMS membrane and then sandwiched the suspension by placing the non-patterned PDMS membrane from the top. The use of the thin PDMS membranes for both top and bottom substrates realized sufficient oxygen supply to the dense bacterial suspension, enabling longer observation of highly active bacterial dynamics and the evaluation of vortex stability.

C. Microscopy

The bright field images of the bacterial dynamics were captured with an inverted microscope (IX83, Evident) equipped with 10x objective lens (UPLSXAPO, NA=0.4) and a large-field-of-view sCMOS camera (Kinetix, Teledyne Photometrics, 3200×3200 pixels, 16 bit). With this setup, we captured a field of view of about 2.1 mm × 2.1 mm with the spatial resolution of 0.65 μm/pixel at 50 fps for 150 seconds amounting 150 GB for a single movie, enabling the simultaneous observation of multiple wells with different radii with 0.5 μm increment (Fig. S1). This allowed us to avoid conducting multiple experiments for different radii using different bacterial cultures, thereby preventing

* nishiguchi@phys.sci.isct.ac.jp

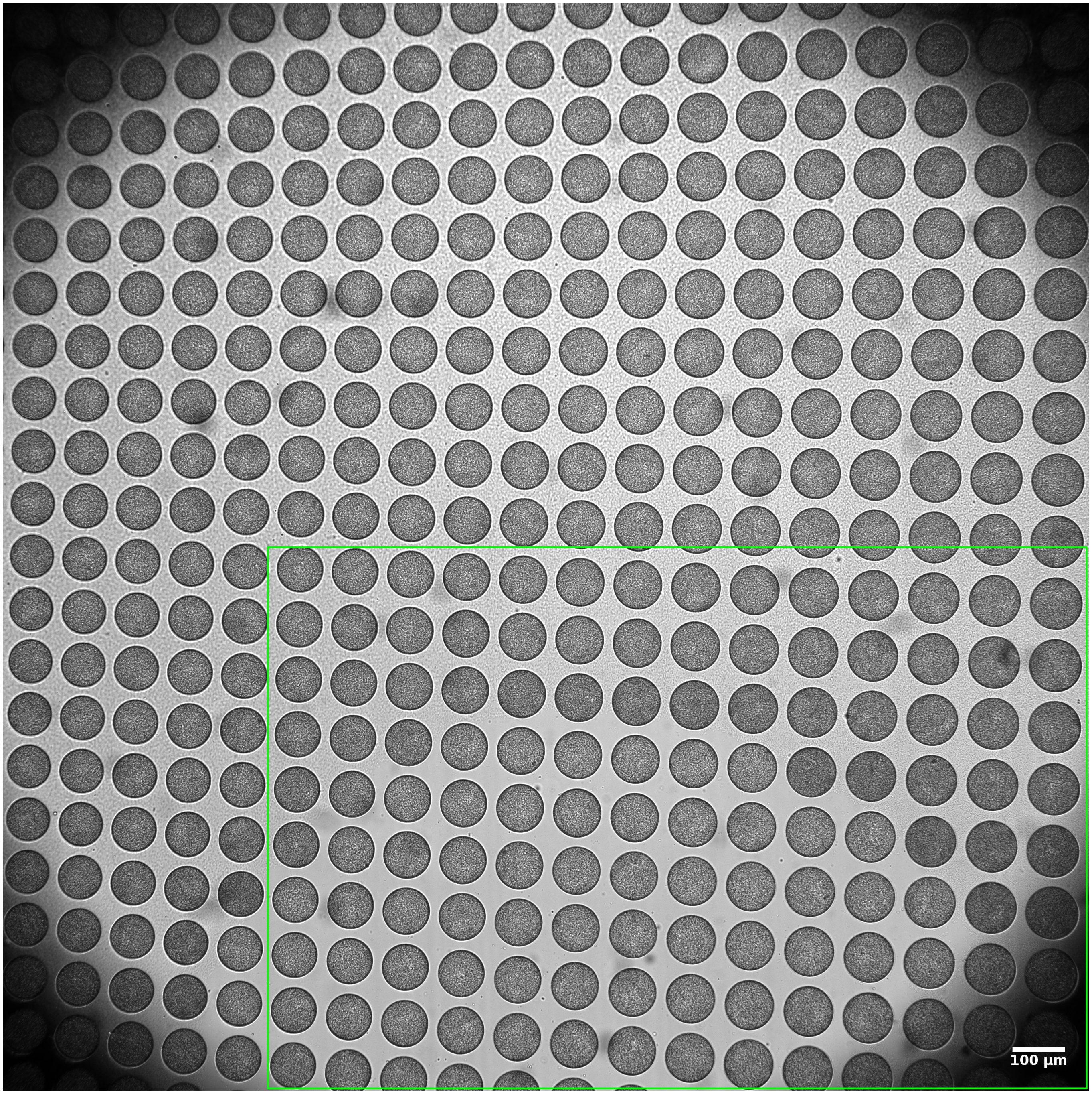

FIG. S1. Snapshot of the whole field of view taken by the camera. The wells aligned in each column have the same designed radii and their radii are increasing from left to right. The wells whose centers were located within the green rectangular region were used for analysis. This rectangular region is displayed in Fig.1(c) in the main text.

uncontrolled variability in the sample states. Because bacterial activity gradually decelerated over a few minutes even within the device made of thin PDMS membranes with high oxygen permeability, the duration of the experimental observation was limited to 150 seconds to prevent nonstationary statistics.

In addition, simultaneous observation of $\sim$ 20 wells with the same designed radii R_{design} was crucial for our purpose. The bacterial density within each well cannot be uniform due to the confinement process, which can be visually confirmed by the brightness of the bright field images (Fig. S1). The slight variations of the density largely affect the dynamics of the bacterial collective motion very close to the transition point. This is the reason why, even among the wells with the same designed radii R_{design}, we observed variations of the spin correlation times [Fig. 2(c)]

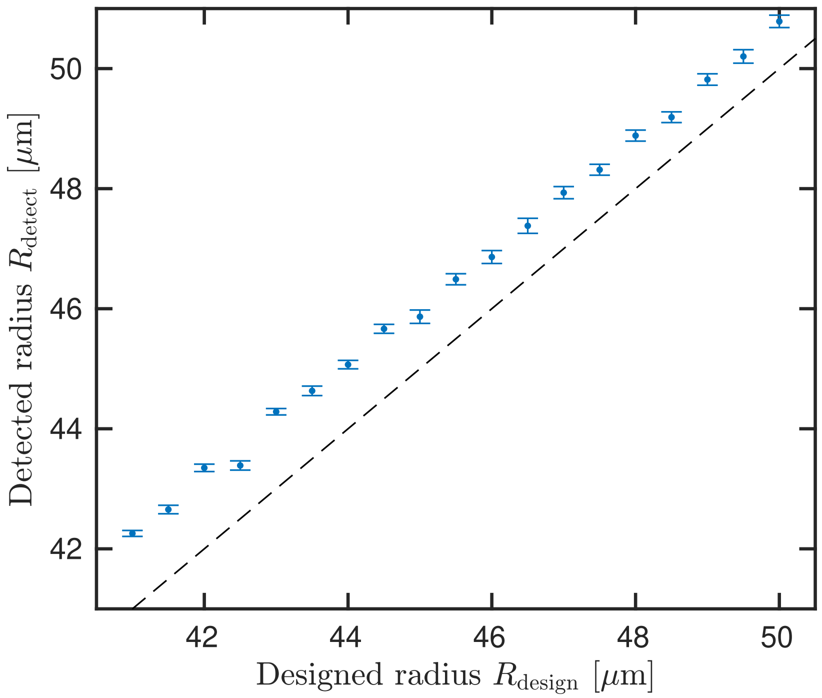

FIG. S2. Comparison between the designed radii R_{design} and the detected radii R_{detect} of the wells. The wells in the field of view (Fig. S1) contained wells with R_{design} ranging from 41.0 to 50.0 μm with 0.5 μm increment. The detected radii R_{detect} are slightly larger than the designed radii R_{design}, with the average difference of 0.98 ± 0.05 μm ($\pm$: standard errors). Blue circles are detection results. A black dashed line is the line $R_{\mathrm{detect}} = R_{\mathrm{design}}$ to guide the eye. Error bars: standard errors.

and the CW-CCW bias in the rotational directions (Fig. S3).

D. Image processing

The obtained movie was processed by using the open-source MATLAB plugin PIVlab [1] to obtain the velocity field $\mathbf{v}_{\mathrm{raw}}(x, y, t)$. The PIV interrogation box size was 16×16 pixels (10.4 μm ×10.4 μm, sufficiently smaller than the length scale of bacterial collective motion) with the step size of 8 pixels (5.2 μm, 50% overlap). Since the use of the PDMS membranes for both substrates instead of glass substrates resulted in the deflection of the device and reduced optical resolutions, we applied a three-dimensional Gaussian filter, $G(x, y, t) = \frac{1}{(2\pi)^{3/2}\sigma_x\sigma_y\sigma_t} \exp\left[-\left(\frac{x^2}{2\sigma_x{}^2} + \frac{y^2}{2\sigma_y{}^2} + \frac{t^2}{2\sigma_t{}^2}\right)\right]$, to the $\mathbf{v}_{\mathrm{raw}}(x, y, t)$ to remove the noise. The standard deviations of the Gaussian were chosen as $\sigma_x = \sigma_y = 16$ pixels (2 PIV grids) and $\sigma_t = 0.04$ s (2 frames), which gave the best results in our experimental conditions. All the data presented in the main text were based on the filtered velocity field, $\mathbf{v}(x, y, t) = \iiint \mathbf{v}_{\mathrm{raw}}(x', y', t') G(x - x', y - y', t - t')\, dx'\, dy'\, dt'$.

The positions and the radii of the wells were detected by the circular Hough transform using the MATLAB function `imfindcircles`. A background image was first obtained by averaging all the 7500 frames of the movie. We applied an adaptive thresholding (contrast limited adaptive histogram equalization, CLAHE) to the background image to cancel the inhomogeneity of the illumination to ensure the accurate detection of the wells. The circular Hough transform was then applied to the processed image to detect the positions and the radii of the wells.

E. Analyzed region and bias in rotational directions

Due to the confinement process, in some regions the bacterial suspensions were not completely confined within the wells and leaked out in between the top and bottom PDMS membranes, which we excluded from the analysis. Since the well patterns were fabricated only on the bottom PDMS membrane, the leakage breaks the up-down symmetry, resulting in weak bias in CW rotations. This is the reason for our choice of the green rectangular region used for the analysis (Fig. S1).

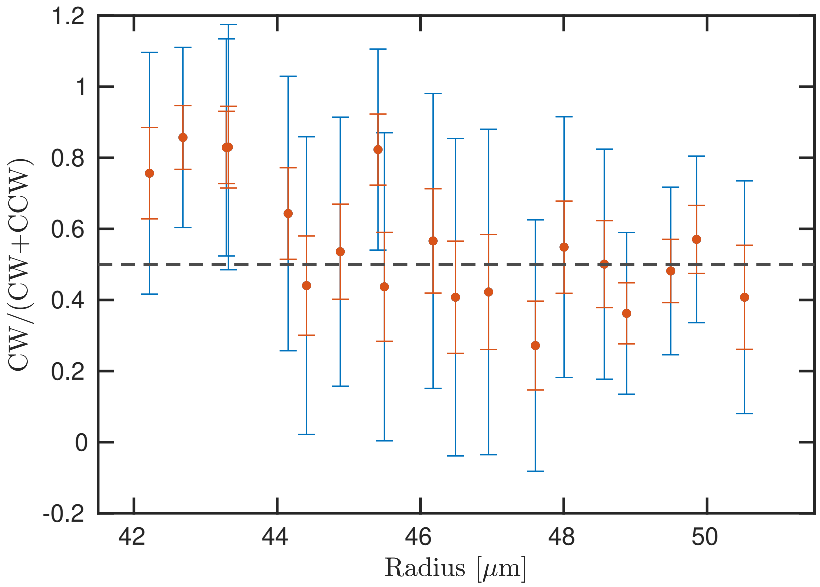

FIG. S3. The fraction of time that the spins rotated in the CW direction, $t_{\mathrm{CW}}/(t_{\mathrm{CW}}+t_{\mathrm{CCW}})$, as a function of the average detected radius $\langle R_{\mathrm{detect}}\rangle$ of the wells with the same designed radius R_{design}. Error bars: standard deviations (blue) and standard errors (red). The wells with the radii < 44 μm showed a significant bias in the CW direction, looking from above, due to the leakage of the bacterial suspension, which was excluded from the analysis.

The bias in CW rotation in the leaked regions looking from the above is exemplified in Fig. S3, in which the wells with the smaller radii on the left side of the green rectangular region in Fig. S1 are also plotted. The bias in the rotational directions was quantified by calculating the fraction of time of CW rotation, $t_{\mathrm{CW}}/(t_{\mathrm{CW}}+t_{\mathrm{CCW}})$, where t_{CW} and t_{CCW} are the durations of CW and CCW rotations judged by the sign of the spin $S_i(t)$, respectively. We calculated this quantity for each well and then calculated the mean value of the wells with the same designed radius R_{design}. The absence of the CW-CCW bias was used as a criterion for determining the regions with good confinement to be used in the further analysis.

F. Correlation time of the spins

Correlation times of the spins were calculated to demonstrate the transition from the stabilized vortex state to the oscillatory state, see Fig. S4. For each well, we calculate the spin $S_i(t)$, where i denotes the index of the well. Then, we calculated the correlation function,

$$C_i(\tau) = \frac{\langle S_i(t)S_i(t+\tau)\rangle_t}{\langle S_i(t)^2\rangle_t}, \tag{S1}$$

where $\langle\ \rangle_t$ means temporal averaging. We defined the correlation time of the spin at which $C_i(\tau)$ becomes smaller than $1/e$ for the first time. Within the experimental observation period of 150 s, some wells did not reverse the sign of $S_i(t)$ and exhibited quite stable behavior. Therefore, we could not define the correlation time for such wells and instead we plotted these wells at the vertical axis denoted as > 150 in Fig. 2(c). Since taking the average is not possible due to these data points with diverging correlation times, we calculated the moving median with the window width of 2 μm. To highlight the discontinuity between the diverging data points at > 150 and those with finite correlation times (< 150), the red line connecting these two regions is plotted with a dashed line in Fig.2(c).

G. Random state for smaller wells and turbulent state for larger wells

Since the height of the cylindrical well was 30 μm, further decreasing the well's radius results in three-dimensional bacterial motion, invalidating the assumption of the two-dimensionality of the observed collective motion. This fact, together with our purpose to resolve the highly fluctuating dynamics around the transition point, constrained our experimental observation in the range of the radii from $R = 44.0$ μm to $R = 50.5.0$ μm as shown in Fig. 1. Nonetheless,

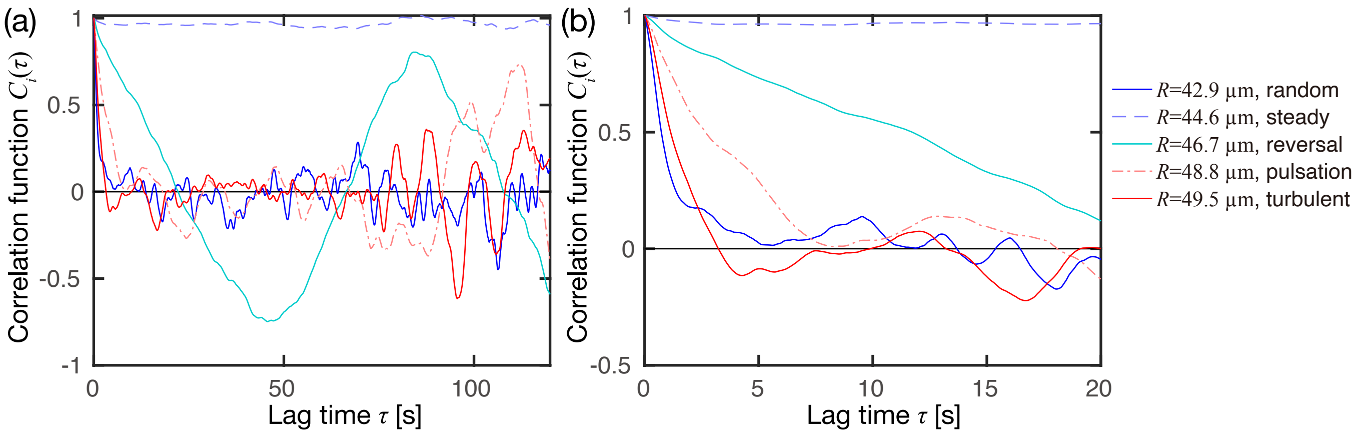

FIG. S4. Correlation functions $C_i(\tau)$ for random motion (solid blue, $R = 42.9$ μm, same as Supplementary Movie 9), a steady single vortex (dashed blue, $R = 44.6$ μm), reversing vortices (solid cyan, $R = 46.7$ μm), pulsating four vortices (dashed red, $R = 48.8$ μm), and a turbulent state (solid red, $R = 49.5$ μm). Both (a) and (b) show the same data but with different ranges for the lag time τ.

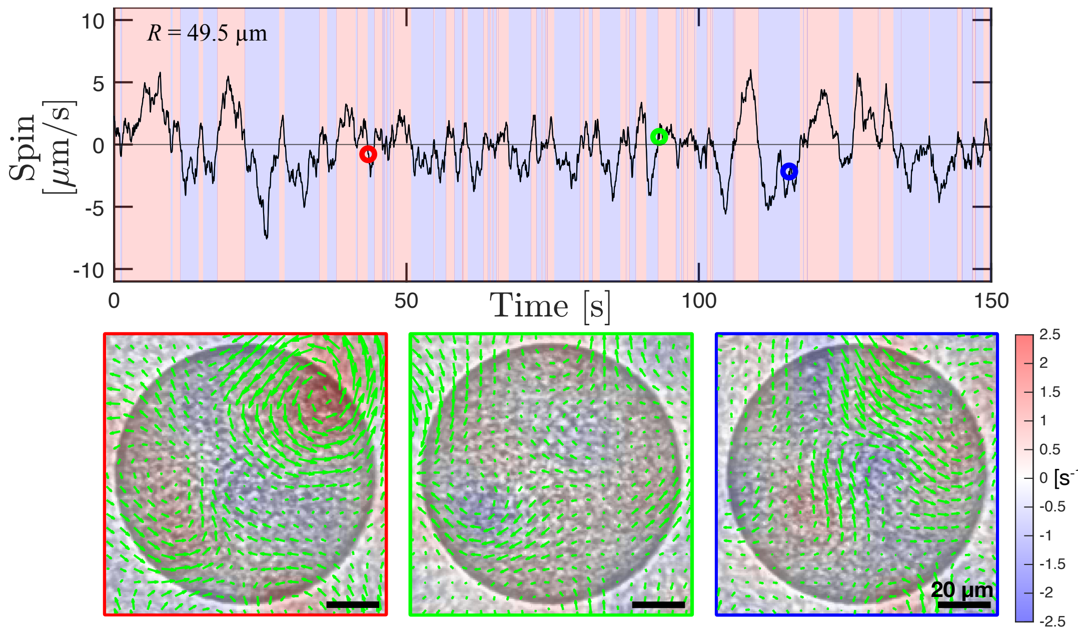

FIG. S5. Time series of a turbulent well with the radius of $R = 49.5$ μm. The instantaneous velocity and vorticity fields are shown below the time series, with the colors of the rectangle corresponding to the time points highlighted by colored circles in the time series (Supplementary Movie 5).

we examined the behavior of bacterial suspension within smaller wells to compare with our theoretical predictions. Our device contains smaller wells than those shown in Fig. S1 outside of this field of view. We observed the dynamics in these wells prior to the observation of the field of view of Fig. S1. The same bacterial suspension was used and the interval between the two successive movies was minimized.

For these sufficiently small wells, we observed that even a single-vortex state was not stably realized, resulting in non-coherent random motion of individual bacteria, see Supplementary Movie 9 and the blue solid lines in Fig. S4. This observation is consistent with our analytical theory, which predicts the presence of a threshold radius for the 1-vortex ($n = 0$) mode to grow, see λ_0 in Fig. 4(a).

When the radius R became larger beyond the pulsating four-vortex regime, we started to observe chaotic dynamics of vortices resembling the bulk active turbulence. An example of such a turbulent well is illustrated in Fig. S5, see Supplementary Movie 5.

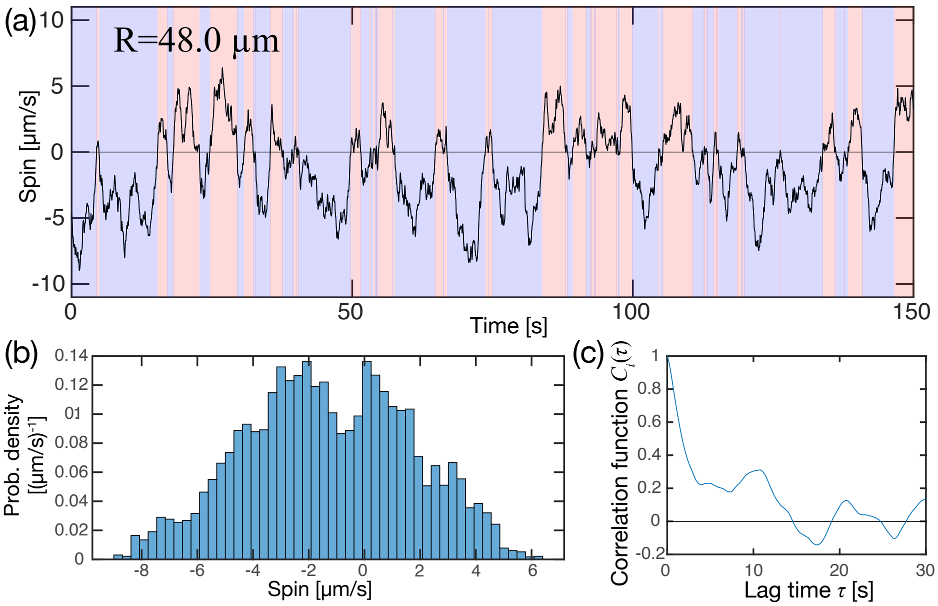

FIG. S6. Spin dynamics in a well with reversing vortices with the radius of $R = 48.0$ μm. (a)Time series of spins. The instantaneous velocity and vorticity fields are shown below the time series, with the colors of the rectangle corresponding to the time points highlighted by colored circles in the time series (Supplementary Movie 10).

H. Reversing vortices with smaller period

In addition to the well exhibiting reversing vortices shown in Fig. 1(c, f), some other wells also exhibit the same properties. In Fig. S6, we provide another example of a well showing vortex reversals with radius $R = 48.0$ μm, which is larger than the reversing well $R = 46.7$ μm but smaller than the pulsating four-vortex state $R = 48.8$ μm in Fig. 1(c, f). As it was closer to the transition point between the single stabilized vortex and the reversing vortices, the interval of the reversals became shorter and faster reversals were observed while keeping the bimodality in the spin distribution (Fig. S6(a,b)), giving faster decay of the correlation function $C_i(\tau)$ and a smaller correlation time (Fig. S6(c)). This is consistent with the infinite period bifurcation scenario revealed in our analytical theory.

I. Velocity & vorticity profiles and fit to the analytical solution

Quantitative comparisons between the experimental data and theoretical solutions were performed by examining the velocity and vorticity profiles for the single stabilized vortex, shown in Fig. 2(a). Due to the PIV interrogation box size 16×16 pixels and the standard deviation of the Gaussian filter $\sigma_x = 16$ pixels, the obtained velocity and vorticity fields can leak out from the radius R detected by the image analysis by 32 pixels, corresponding to 20.8 μm. This length scale is consistent with what is observed in Fig. 2(a), where the experimental data extend beyond the actual radius R depiced as the vertical dashed line. Such treatments were indispensable for processing the images acquired at low magnification, which was essential for statistical analysis that incorporated hundreds of wells in a single field of view to resolve the fluctuating dynamics near the transition point. These penetrating fields may justify the numerical implementation of the damping terms for the velocity and vorticity fields.

The velocity and vorticity profiles in the single stabilized vortex, shown in Fig. 2(a), were fitted by the theoretical curves Eq. (S15) and Eq. (S19) for $n = 0$. The best fits yield $G_{0+} = 1.51$ s^{-1}, $G_{0-} = 0.588$ s^{-1}, $k_{0+} = 0.0914$ μm^{-1}, $k_{0-} = 0.0453$ μm^{-1} for $\mathbf{v}$ and $G_{0+} = 1.31$ s^{-1}, $G_{0-} = 0.633$ s^{-1}, $k_{0+} = 0.0920$ μm^{-1}, $k_{0-} = 0.0483$ μm^{-1} for ω.

II. SUPPLEMENTARY NOTE 2. REGRESSION TO THE TTSHE

A. Least squares method

We inferred the parameters in the TTSHE by regressing the experimental data to the TTSHE. Specifically, we applied the least squares method to the experimental data of the vorticity field $\omega(x, y, t)$ to find the parameters A, C, Γ_0, Γ_2 in the TTSHE. By assuming the vorticity equation for the TTSHE,

$$\lambda(\mathbf{v} \cdot \nabla)\omega + A\omega + C\nabla \times |\mathbf{v}|^2\mathbf{v} - \Gamma_0\nabla^2\omega + \Gamma_2\nabla^4\omega = -\partial_t\omega, \tag{S2}$$

we constructed its matrix representation by using the experimental data. For a single regression, we used the data from time t to $t + (N-1)\Delta t$ with Δt being the time interval between the frames and N being the number of frames used for the regression. The matrix representation is given by,

$$\Phi P = \Psi, \tag{S3}$$

with,

$$P = \begin{pmatrix} \lambda \\ A \\ C \\ \Gamma_0 \\ \Gamma_2 \end{pmatrix}, \quad \Psi = \begin{pmatrix} -\partial_t\omega_t^{\mathbf{r}_1} \\ -\partial_t\omega_t^{\mathbf{r}_2} \\ \vdots \\ -\partial_t\omega_t^{\mathbf{r}_M} \\ -\partial_t\omega_{t+\Delta t}^{\mathbf{r}_1} \\ -\partial_t\omega_{t+\Delta t}^{\mathbf{r}_2} \\ \vdots \\ -\partial_t\omega_{t+\Delta t}^{\mathbf{r}_M} \\ \vdots \\ -\partial_t\omega_{t+(N-1)\Delta t}^{\mathbf{r}_M} \end{pmatrix}, \tag{S4}$$

$$\Phi = \begin{pmatrix} (\mathbf{v}_t^{\mathbf{r}_1} \cdot \nabla)\omega_t^{\mathbf{r}_1} & \omega_t^{\mathbf{r}_1} & \nabla \times |\mathbf{v}_t^{\mathbf{r}_1}|^2\mathbf{v}_t^{\mathbf{r}_1} & -\nabla^2\omega_t^{\mathbf{r}_1} & \nabla^4\omega_t^{\mathbf{r}_1} \\ (\mathbf{v}_t^{\mathbf{r}_2} \cdot \nabla)\omega_t^{\mathbf{r}_2} & \omega_t^{\mathbf{r}_2} & \nabla \times |\mathbf{v}_t^{\mathbf{r}_2}|^2\mathbf{v}_t^{\mathbf{r}_2} & -\nabla^2\omega_t^{\mathbf{r}_2} & \nabla^4\omega_t^{\mathbf{r}_2} \\ \vdots & \vdots & \vdots & \vdots & \vdots \\ (\mathbf{v}_t^{\mathbf{r}_M} \cdot \nabla)\omega_t^{\mathbf{r}_M} & \omega_t^{\mathbf{r}_M} & \nabla \times |\mathbf{v}_t^{\mathbf{r}_M}|^2\mathbf{v}_t^{\mathbf{r}_M} & -\nabla^2\omega_t^{\mathbf{r}_M} & \nabla^4\omega_t^{\mathbf{r}_M} \\ (\mathbf{v}_{t+\Delta t}^{\mathbf{r}_1} \cdot \nabla)\omega_{t+\Delta t}^{\mathbf{r}_1} & \omega_{t+\Delta t}^{\mathbf{r}_1} & \nabla \times |\mathbf{v}_{t+\Delta t}^{\mathbf{r}_1}|^2\mathbf{v}_{t+\Delta t}^{\mathbf{r}_1} & -\nabla^2\omega_{t+\Delta t}^{\mathbf{r}_1} & \nabla^4\omega_{t+\Delta t}^{\mathbf{r}_1} \\ (\mathbf{v}_{t+\Delta t}^{\mathbf{r}_2} \cdot \nabla)\omega_{t+\Delta t}^{\mathbf{r}_2} & \omega_{t+\Delta t}^{\mathbf{r}_2} & \nabla \times |\mathbf{v}_{t+\Delta t}^{\mathbf{r}_2}|^2\mathbf{v}_{t+\Delta t}^{\mathbf{r}_2} & -\nabla^2\omega_{t+\Delta t}^{\mathbf{r}_2} & \nabla^4\omega_{t+\Delta t}^{\mathbf{r}_2} \\ \vdots & \vdots & \vdots & \vdots & \vdots \\ (\mathbf{v}_{t+(N-1)\Delta t}^{\mathbf{r}_1} \cdot \nabla)\omega_{t+(N-1)\Delta t}^{\mathbf{r}_1} & \omega_{t+(N-1)\Delta t}^{\mathbf{r}_M} & \nabla \times |\mathbf{v}_{t+(N-1)\Delta t}^{\mathbf{r}_1}|^2\mathbf{v}_{t+(N-1)\Delta t}^{\mathbf{r}_1} & -\nabla^2\omega_{t+(N-1)\Delta t}^{\mathbf{r}_1} & \nabla^4\omega_{t+(N-1)\Delta t}^{\mathbf{r}_1} \\ \vdots & \vdots & \vdots & \vdots & \vdots \\ (\mathbf{v}_{t+(N-1)\Delta t}^{\mathbf{r}_M} \cdot \nabla)\omega_{t+(N-1)\Delta t}^{\mathbf{r}_M} & \omega_{t+(N-1)\Delta t}^{\mathbf{r}_M} & \nabla \times |\mathbf{v}_{t+(N-1)\Delta t}^{\mathbf{r}_M}|^2\mathbf{v}_{t+(N-1)\Delta t}^{\mathbf{r}_M} & -\nabla^2\omega_{t+(N-1)\Delta t}^{\mathbf{r}_M} & \nabla^4\omega_{t+(N-1)\Delta t}^{\mathbf{r}_M} \end{pmatrix}, \tag{S5}$$

where $\mathbf{r}_1$ to $\mathbf{r}_M$ denote the positions within the region of interest (ROI) used for the regression, and $\mathbf{v}_t^{\mathbf{r}_k} = \mathbf{v}(\mathbf{r}_k, t)$ and $\omega_t^{\mathbf{r}_k} = \omega(\mathbf{r}_k, t)$. Each column of Φ and Ψ contains data from all the M positions within the ROI and all the time points between t and $t + (N-1)\Delta t$, totaling MN elements. This overdetermined system was solved by the least squares method using the pseudo-inverse matrix of Φ defined as,

$$\Phi^+ = (\Phi^T\Phi)^{-1}\Phi^T, \tag{S6}$$

where Φ^T is the transpose of Φ. Then, the optimal parameters P_{opt} were estimated as,

$$P_{\text{opt}} = \Phi^+\Psi. \tag{S7}$$

Since the TTSHE is a model for the bulk flow, we excluded the region close to the confining boundary from the ROI so that the boundary does not affect the parameter estimations. The time window $N\Delta t$ was determined based on the spin correlation time. We repeated this regression at time t and then shifted the time window to obtain the time series of estimated parameters. In this computation, we used central differences except for $\nabla^2\omega$ and $\nabla^4\omega$ that were calculated by using the fast Fourier transform.

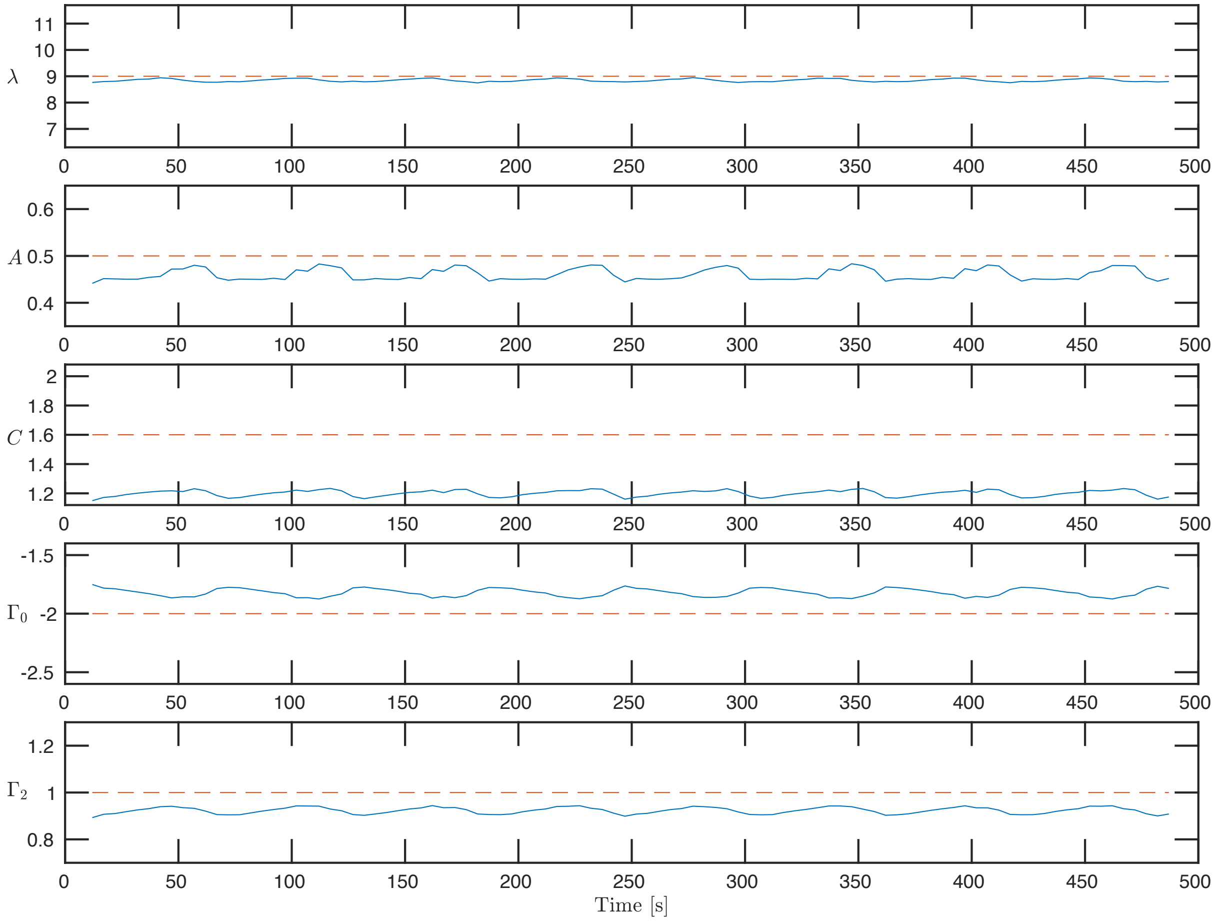

FIG. S7. Time series of the parameters in the TTSHE estimated from the regression of the numerical data. Blue curves are the results of the regression, and the red dashed lines are the ground truth values used for generating the numerical dataset. The vertical axes in all the plots ranges from -30% to +30% of the ground truth values.

B. Verification with numerical data set

To verify this method, we first applied this regression to the numerical data obtained from the TTSHE. We used the data for $R = 5.30$, which shows the two oscillating vortices with the period of 117 in the numerical unit. A circular ROI centered at the well with half of the well's radius $R/2 = 2.65$ was chosen to diminish the boundary effects. The time window was chosen to be $N\Delta t = 22$, which is the correlation time of this oscillatory state. To be consistent with experimental data, we decreased the spatial resolution of our high-resolution numerical data by 8 folds in both x and y directions, resulting in spatial resolution of $\Delta x = 0.32$, and Δt was set to 1. These settings result in $M = 217$ and $N = 22$, and the duration of the numerical data was 500. To estimate the standard errors of the average values, we used the effective number of independent samples as $N_{\text{sample}} = 500/22 = 22.7$, which we calculated on the basis of the time window $N\Delta t = 22$ s and the duration of the data 500.

As shown in Fig. S7 and in Table S1, the time series of the estimated parameters fluctuate in time but their temporal averages gave reasonable values. Remarkably, the most accurate estimation was achieved for the most important parameter λ, which determines the strength of the nonlinear advection and the onset of the limit cycle. The error of the estimated λ was as small as 2%, while those of other parameters were 8% to 25%. Therefore, this estimation method can reliably be applied at least to estimating λ. The estimations of the other parameters have difficulties arising from their nonlinearities and higher-order derivatives.

TABLE S1. Estimated parameters of the TTSHE from the regression of the numerical data.

	λ	A	C	Γ_0	Γ_2
Ground truth	9	0.5	1.6	-2	1
Average	8.844	0.4599	1.2033	-1.8204	0.9231
Standard error	0.012	0.0026	0.0046	0.0073	0.0030
Standard deviation	0.054	0.012	0.021	0.034	0.014

TABLE S2. Estimated parameters in the TTSHE from the regression of the experimental data. The brackets [ ] indicate the units of the parameters. The time-averaged values have signs consistent with our numerical parameters. Note that λ is nondimensional.

	λ	A [s^{-1}]	C [$\mu\mathrm{m}^{-2}\cdot\mathrm{s}$]	Γ_0 [$\mu\mathrm{m}^2\cdot\mathrm{s}^{-1}$]	Γ_2 [$\mu\mathrm{m}^4\cdot\mathrm{s}^{-1}$]
Average	1.69	0.124	0.00011	-13.8	2.5
Standard error	0.12	0.047	0.00022	3.9	23
Standard deviation	0.38	0.14	0.00068	11.9	71

C. Application to experimental data

Now we apply this regression to our experimental data. We used the velocity and vorticity field data within the well with oscillatory behavior presented in Fig. 1(f). We excluded the region within 32 pixels (20.8 μm) from the boundary of the well, which composed the ROI for the regression. The choice of 32 pixels was based on the sum of the PIV interrogation box size 16×16 pixels and the standard deviation of the Gaussian filter $\sigma_x = 16$ pixels. Above this length scale, the estimated PIV velocity field is not affected by the boundary. With this choice, our ROI contained $M = 107$ positions.

The time window $N\Delta t$ used for the regression was determined on the basis of the spin correlation time. Since the spin correlation time for this well was 14.2 s, which corresponds to 710 frames, we chose $N = 710$.

We obtained the time series of estimated parameters shown in Fig. S8. Based on the time window $N\Delta t = 14.2$ s and the duration of the movie 150 s, we estimate the effective number of independent samples as $N_{\mathrm{sample}} = 150/14.2 = 10.6$, which is then used for evaluating the standard errors of the estimated parameters. The average values and the standard errors of the estimated parameters are shown in Table S2. As expected, the regression gave all the signs of the estimated parameters consistent with the numerical parameters in the main text.

Although the standard errors of the estimation were relatively large for the nonlinear term C and the fourth-order derivative term Γ_2, the advection term λ is rather reliably estimated, as expected from our control calculation with the numerical data set. The value $\lambda = 1.69 \pm 0.12$, which is larger than unity, is indeed a characteristic of the active turbulence model of pusher-type microswimmers such as *Bacillus subtilis*. To compare with the nondimensional form of the TTSHE, we need to choose typical length L, time T and velocity V. After nondimensionalization, all the coefficients scale as $\lambda \to \frac{VT}{L}\lambda$, $A \to TA$, $C \to V^2TC$, $\Gamma_0 \to \frac{T}{L^2}\Gamma_0$, and $\Gamma_2 \to \frac{T}{L^4}\Gamma_2$. Therefore, if we choose a large value for V, e.g. based on an ideal unconstrained bacterial turbulence without confinement to the well, the value of λ can be in the range where we found a limit cycle in our analytical calculation as we describe in the main text.

D. Regression to other equations

The use of the TTSHE was justified on the basis of the regression to another model, Nikolaevskiy equation [2, 3], also used as a model for the fluid motion of dense bacterial suspensions. Nikolaevskiy equation is, together with the incompressibility, given by,

$$\nabla \cdot \mathbf{v} = 0, \tag{S8}$$

$$\partial_t \mathbf{v} + (\mathbf{v}\cdot\nabla)\mathbf{v} = -\nabla p + \Gamma_0 \nabla^2 \mathbf{v} - \Gamma_2 \nabla^4 \mathbf{v} + \Gamma_4 \nabla^6 \mathbf{v}. \tag{S9}$$

We rewrote these equations into a vorticity equation by taking the rotation,

$$\Gamma_0 \nabla^2 \omega - \Gamma_2 \nabla^4 \omega + \Gamma_4 \nabla^6 \omega = \partial_t \omega + (\mathbf{v}\cdot\nabla)\omega, \tag{S10}$$

and reconstructed a matrix representation for Nikolaevskiy equation for regression, in the same way as we did for the TTSHE. We obtained the optimal parameters as shown in Table S3, with physically reasonable signs, $\Gamma_0 > 0$, $\Gamma_2 < 0$, and $\Gamma_4 > 0$, for active turbulence with a characteristic length scale.

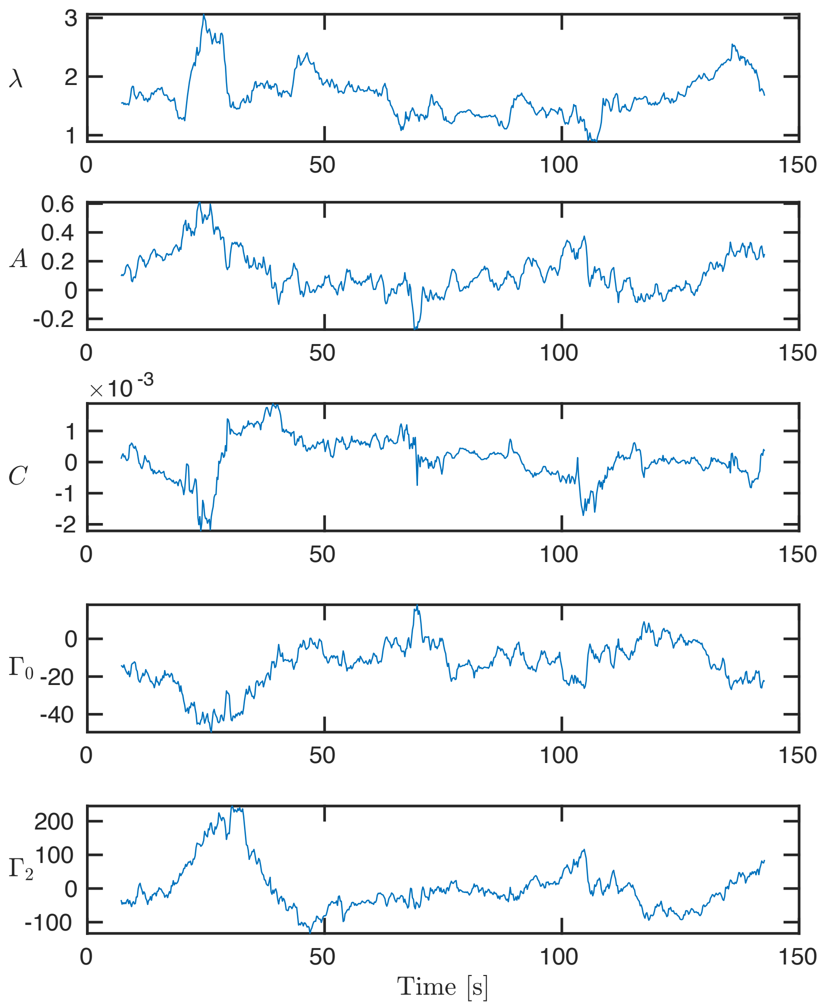

FIG. S8. Estimated parameters in the TTSHE from the regression of the experimental data as functions of time.

TABLE S3. Estimated parameters in the Nikolaevskiy equation from the regression of the experimental data. The brackets [ ] indicate the units of the parameters.

	Γ_0 [μm$^2 \cdot$ s^{-1}]	Γ_2 [μm$^4 \cdot$ s^{-1}]	Γ_4 [μm$^6 \cdot$ s^{-1}]
Average	11.5	-803.7	2808.8
Standard error	2.6	135.0	662.5
Standard deviation	8.0	417.4	2048.7

We evaluated the performance of the two models by comparing the residuals of the regression, R_{T} for the TTSHE and R_{N} for Nikolaevskiy equation, respectively. Specifically, we calculated,

$$R_{\mathrm{T}} = \|\Phi P_{\mathrm{opt}} - \Psi\|^2, \tag{S11}$$

where $\| \ \|$ denotes the norm of a vector. We compute these residuals for each time point t. The same procedure was applied also to Nikolaevskiy equation, and the time series of both models are plotted in Fig. S9. As a result, R_{N} is always larger than R_{T}, suggesting that the TTSHE outperforms Nikolaevskiy equation in terms of their ability to describe bacterial turbulence, at least, in our setup.

We note that it would be ideal to try modern machine learning techniques such as SINDy [4] to infer the hydrodynamic description for bacterial turbulence by preparing the library of the possible terms. However, this is beyond the scope of our current work.

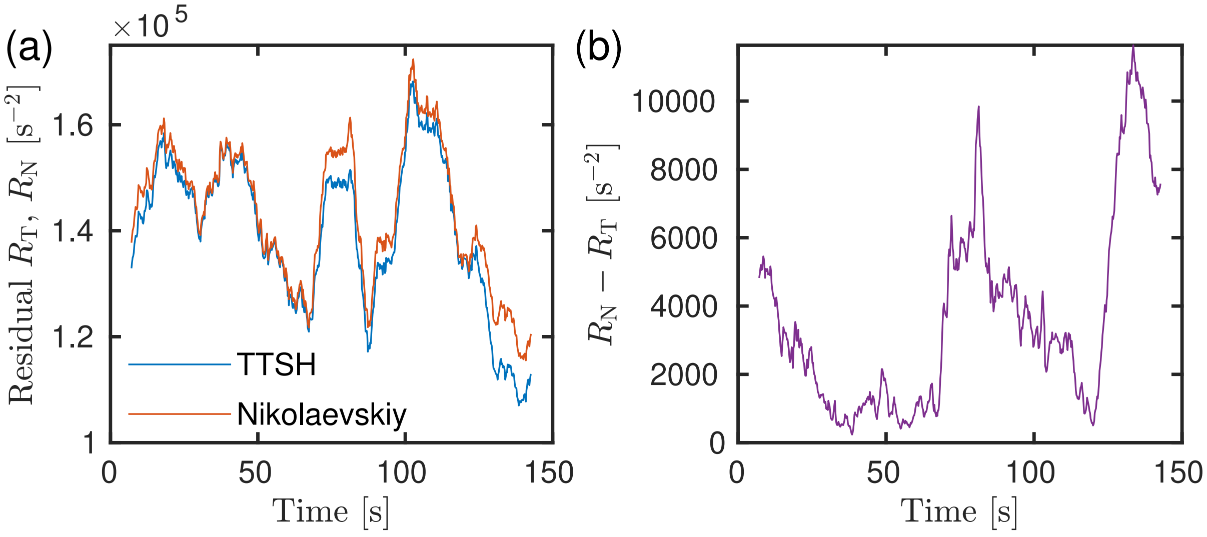

FIG. S9. Residuals of the regressions. (a) Time series of the residuals for TTSHE R_T (blue) and Nikolaevskiy equation R_N (red). (b) Time series of the difference of the residuals $R_\mathrm{N} - R_\mathrm{T}$.

III. SUPPLEMENTARY NOTE 3. DETAILS OF COMPUTATIONAL MODELING

A. Model with Boundary

To incorporate the effect of boundary, we use the following equation [5, 6]:

$$\frac{\partial \omega}{\partial t} + \lambda \mathbf{v} \cdot \boldsymbol{\nabla} \omega = a\omega - b\boldsymbol{\nabla} \times \left[|\mathbf{v}|^2 \mathbf{v}\right] - \left(1 + \nabla^2\right)^2 \omega - \gamma_\mathbf{v} \boldsymbol{\nabla} \times [K(\mathbf{r})\mathbf{v}] - \gamma_\omega K(\mathbf{r})\omega, \tag{S12}$$

where $K(\mathbf{r})$ is a positive-valued scalar field introduced to damp $\mathbf{v}, \omega$ outside the well, and $\gamma_\mathbf{v}, \gamma_\omega > 0$ are the associated damping strengths. From its design, K is defined to be $\simeq 0$ inside the well while $\simeq 1$ for the outside. Similar to the model parameters, we also need to determine the values of $\gamma_\mathbf{v}, \gamma_\omega$ based on experiments. In this paper, we adopt the choice $(\lambda, a, b, \gamma_\mathbf{v}, \gamma_\omega) = (9, 0.5, 1.6, 40, 4)$ suggested in Ref. [6].

B. Simulation Scheme

To solve Eq. (S12), we use the pseudospectral method [6], which goes back and forth between the real and Fourier space to compute the spatial derivatives. In this algorithm, the real space coordinates are defined on a periodic grid $\mathbf{r} \in \Delta x \cdot \mathbb{Z}^2_{[0,N-1]}$ where N is the number of grid points per dimension and Δx is the grid spacing. For the Fourier space, wavenumber vector $\mathbf{k}$ resides in $(\pi/L) \cdot [-N, N]^2$ where $L = N\Delta x$. Each time we encounter spatial derivatives, we first Fourier transform quantities using the discrete Fourier transform (DFT), substitute $\boldsymbol{\nabla}$ with $i\mathbf{k}\cdot$, and then push the result back to the real space by the inverse DFT (iDFT). The time evolution is done by the Euler method in the Fourier space since we can exactly handle the linear terms in a stable manner (operator splitting). After updating ω (from time t to $t + \Delta t$), we compute the associated incompressible velocity field $\mathbf{v}$ by the streamfunction method, which first solves the Poisson equation $\nabla^2 \psi = -\omega$ and then computes $\hat{\mathbf{v}}_x = i\mathbf{k}_y \hat{\psi}$, $\hat{\mathbf{v}}_y = -i\mathbf{k}_x \hat{\psi}$ where $\hat{*}$ denotes the Fourier transform. As this method does not fix the average velocity $\hat{\mathbf{v}}(\mathbf{0})$, we need to compute the dynamics of them separately.

In addition to the procedures described above, we need to take care of two simulation-specific issues. First, we cannot compute the full dynamics for all the $\mathbf{k}$ because real-space multiplication doubles the range of $\mathbf{k}$. To deal with that, we manually discard the high-$\mathbf{k}$ modes before and after real-space multiplications. Similar treatment is also applied to K. Specifically, we use the following procedure to generate appropriate K from the naive boolean mask $K_\mathrm{naive}(\mathbf{r}) = 0$ if $|\mathbf{r}| \le R,\ 1$ otherwise:

Algorithm 1 smoothing K

Input: K_{naive}

$\hat{K}_{\text{naive}} = \text{DFT}[K_{\text{naive}}]$
for all k do
  if $\mathbf{k}^2 > \left(\frac{1}{4}\cdot\frac{\pi}{\Delta x}\right)^2$ **then**
    $\hat{K}_{\text{naive}}(\mathbf{k}) = 0$
  end if
end for
$K = \text{iDFT}[\hat{K}_{\text{naive}}]^2$

Output: processed $K(\mathbf{r})$

Note that the positivity of K is guaranteed by the square operation. Second, numerical errors can lead to the violation of $\omega(\mathbf{k}) = \omega(-\mathbf{k})^*$, which is a necessary condition for the real-valued ω. To enforce this condition, we symmetrize ω each time after the Euler update.

Putting everything together, the simulation proceeds as follows:

Algorithm 2 simulation scheme

Input: $(\mathbf{v}(t),\ \hat{\omega}(t))$

$\mathbf{rhs}_1 = -b\mathbf{v}^2\mathbf{v} - \gamma_{\mathbf{v}}K\mathbf{v}$
$\text{rhs}_2 = -\lambda\mathbf{v}\cdot\text{iDFT}[i\mathbf{k}\hat{\omega}] - \gamma_\omega K\omega$
$\hat{\text{rhs}} = i\mathbf{k}\times\hat{\mathbf{rhs}}_1 + \hat{\text{rhs}}_2$
$\hat{\omega}_{\text{naive}} = \exp\left[\left\{a - \left(1-\mathbf{k}^2\right)^2\right\}\Delta t\right]\left(\hat{\omega} + \hat{\text{rhs}}\Delta t\right)$
for all k do
  if $\mathbf{k} = \mathbf{0}$ **then**
    $\hat{\omega}_{\text{new}}(\mathbf{0}) = 0$
    $\hat{\mathbf{v}}_{\text{new}}(\mathbf{0}) = \exp\left[(a-1)\Delta t\right]\cdot\sum_{\mathbf{r}}\frac{1}{\sqrt{N^2}}(\mathbf{v} + \mathbf{rhs}_1\Delta t)$
  else if $\mathbf{k}^2 > \left(\frac{1}{2}\cdot\frac{\pi}{\Delta x}\right)^2$ **then**
    $\hat{\omega}_{\text{new}}(\mathbf{k}) = 0$
    $\hat{\mathbf{v}}_{\text{new}}(\mathbf{k}) = \mathbf{0}$
  else
    $\hat{\omega}_{\text{new}}(\mathbf{k}) = \frac{1}{2}\{\hat{\omega}_{\text{naive}}(\mathbf{k}) + \hat{\omega}_{\text{naive}}(-\mathbf{k})^*\}$
    $\hat{\psi}(\mathbf{k}) = \frac{\hat{\omega}_{\text{new}}(\mathbf{k})}{\mathbf{k}^2}$
    $\hat{\mathbf{v}}_{\text{new}}(\mathbf{k})|_x = i\mathbf{k}_y\hat{\psi}(\mathbf{k}),\ \hat{\mathbf{v}}_{\text{new}}(\mathbf{k})|_y = -i\mathbf{k}_x\hat{\psi}(\mathbf{k})$
  end if
end for

Output: $(\mathbf{v}_{\text{new}},\ \hat{\omega}_{\text{new}}) = (\mathbf{v}(t+\Delta t),\ \hat{\omega}(t+\Delta t))$

In typical simulations, we set $N = 8192$, $\Delta x = 0.005$, and $\Delta t = 0.01$. All the computations were performed on GPUs (NVIDIA RTX A6000 or A100) in single precision, which achieves ~ 60 times speedup compared to CPU implementation with standard CPUs (e.g. Intel Xeon W-2295) [7].

IV. SUPPLEMENTARY NOTE 4. ANALYTICAL THEORY

A. Linear Theory

We start with the TTSHE in the form

$$\partial_t\omega + \lambda\mathbf{v}\cdot\nabla\omega = a\omega - (1+\nabla^2)^2\omega - b\nabla\times|\mathbf{v}|^2\mathbf{v}. \tag{S13}$$

Consider a linearized Eq. (S13) in a disk domain of a radius R

$$\partial_t\omega = a\omega - (1+\nabla^2)^2\omega. \tag{S14}$$

A generic solution to Eq. (S14) can be written in the form $\omega = \sum_{n=-\infty}^{\infty}\omega_n$,

$$\omega_n = \exp(\lambda_n t)\left(G_{n+}J_n(k_{n+}r) + G_{n-}J_n(k_{n-}r)\right)\exp(in\theta), \tag{S15}$$

where λ_n is the growth rate, $G_{n\pm}$ arbitrary constants, J_n are the Bessel functions, and $k_{n\pm}$ are given by the equation

$$k_{n\pm} = \sqrt{1 \pm \sqrt{a - \lambda_n}}. \tag{S16}$$

First, we calculate the stream function ψ satisfying the condition $\nabla^2\psi = -\omega$. In polar coordinates, for each azimuthal mode n we obtain

$$\frac{1}{r}\partial_r(r\partial_r\psi_n) - \frac{n^2}{r^2}\psi_n = -\omega_n. \tag{S17}$$

The solution is

$$\psi_n = \left(\frac{G_{n+}}{k_{n+}^2}J_n(k_{n+}r) + \frac{G_{n-}}{k_{n-}^2}J_n(k_{n-}r) + G_{n0}r^n\right)\exp(in\theta) + \text{c.c.} \tag{S18}$$

Here, the term $G_{n0}r^n$ is a solution to the Laplace equation in polar coordinates $\frac{1}{r}\partial_r(r\partial_r\psi_n) - \frac{n^2}{r^2}\psi_n = 0$.

Correspondingly, we obtain for the velocity components

$$v_\theta = -\partial_r\psi_n = -\left(\frac{G_{n+}}{2k_{n+}}(J_{n-1}(k_{n+}k_r) - J_{n+1}(k_{n+}r)) + \frac{G_{n-}}{2k_{n-}}(J_{n-1}(k_{n-}r) - J_{n+1}(k_{n-}r)) + nG_{n0}r^{n-1}\right)\exp(in\theta) + \text{c.c.}, \tag{S19}$$

$$v_r = \frac{1}{r}\partial_\theta\psi_n = \frac{in}{r}\left(\frac{G_{n+}}{k_{n+}^2}J_n(k_{n+}r) + \frac{G_{n-}}{k_{n-}^2}J_n(k_{n-}r) + G_{n0}r^n\right)\exp(in\theta) + \text{c.c.} \tag{S20}$$

Now, to satisfy the b.c., we have the following conditions,

$$G_{n+}J_n(k_{n+}R) + G_{n-}J_n(k_{n-}R) = 0, \tag{S21}$$

$$\frac{G_{n+}}{k_{n+}^2}J_n(k_{n+}R) + \frac{G_{n-}}{k_{n-}^2}J_n(k_{n-}R) + G_{n0}R^n = 0, \tag{S22}$$

$$\frac{G_{n+}}{2k_{n+}}(J_{n-1}(k_{n+}R) - J_{n+1}(k_{n+}R)) + \frac{G_{n-}}{2k_{n-}}(J_{n-1}(k_{n-}R) - J_{n+1}(k_{n-}R)) + nG_{n0}R^{n-1} = 0. \tag{S23}$$

We can set $G_{n+} = 1$ due to system linearity. Then, the above 3 equations have 3 unknowns $(\lambda_n, G_{n-}, G_{n0})$. It provides the following characteristic equation to determine the growth rates λ_n for arbitrary n:

$$\frac{J_{n-1}(k_{n+}R)}{k_{n+}J_n(k_{n+}R)} - \frac{J_{n-1}(k_{n-}R)}{k_{n-}J_n(k_{n-}R)} - \frac{2n}{R}(1/k_{n+}^2 - 1/k_{n-}^2) = 0 \tag{S24}$$

B. Orthogonality condition

Equation for the eigenmodes and eigenvalues

$$\hat{L}\omega_n = (a - (1 + \nabla_n^2)^2)\omega_n = \lambda_n\omega_n \tag{S25}$$

has rather subtle features (here $\nabla_n^2 = r^{-1}\partial_r r\partial r - n^2/r^2$ is the radial Laplacian). Namely, while the differential operator is symmetric, it is not formally self-adjoint since the orthogonality condition can not be applied to the vorticity modes ω_n. For the proper self-adjointness, the boundary conditions (b.c.) need to be explicit functions of the vorticity ω and its derivatives. Because part of the b.c. $\mathbf{v} = \mathbf{0}$ is expressed in terms of the velocity, the partial integration in the scaler product of the eigenfunctions leads to non-vanishing boundary terms. By formulating the equations in terms of the stream function, the b.c can be reformulated in terms of the derivatives of the stream function, which leads to zero boundary terms in the scalar product of the eigenfunctions. If we consider a scalar product,

$$\langle\omega_n^k\omega_n^l\rangle \neq 0, \tag{S26}$$

the eigenmodes are not orthogonal. Here $\omega_n^{l,k}$ are different modes corresponding to the same n. It can be seen from examining the product $\langle\omega_n^k\hat{L}\omega_n^l\rangle$ that generates non-vanishing boundary terms after integration by parts. In the standard situation, the boundary terms vanish due to the boundary conditions on ω. But we have instead boundary conditions for the stream function ψ. It implies that the orthogonality should be implemented differently, for the stream function ψ_n rather than the vorticity.

Therefore, we formulate the eigenvalue problem in terms of the stream function

$$\hat{L}_1\psi_n = (a - (1+\nabla_n^2)^2)\nabla_n^2\psi_n = \lambda_n\nabla_n^2\psi_n. \tag{S27}$$

The b.c. are $\psi_n = 0, \partial_r\psi_n = 0, \partial_r^2\psi_n = 0$ for $r = R$. With this b.c., the operator $\hat{L}_1$ is self-adjoint, and the boundary terms vanish identically. A little subtlety here is that it is a generalized eigenvalue problem because instead of $\lambda_n\psi_n$ we have $\lambda_n\nabla_n^2\psi_n$.

Now consider the orthogonality condition for each ψ_n mode. The inhomogeneous equation is written as

$$\hat{L}_1 w = (a - (1+\nabla_n^2)^2)\nabla_n^2 w = -\partial_t\omega - \nabla\times\mathbf{B}, \tag{S28}$$

where $\mathbf{B} = -b|\mathbf{v}|^2\mathbf{v} - \lambda\mathbf{v}\cdot\nabla\mathbf{v}$. The solvability means that the r.h.s. is orthogonal to the zero eigenmodes ψ_n. Applying the solvability condition, after partial integration we obtain

$$\int_0^R d\theta r\psi^*\nabla\times\mathbf{B}dr = \int_0^R r\mathbf{v}^*\mathbf{B}dr, \tag{S29}$$

where $\mathbf{v} = (v_r, v_\theta)$, where $v_r = \partial_\theta\psi/r, v_\theta = -\partial_r\psi$. Correspondingly, for the normalization coefficients, we obtain, after partial integration,

$$\int_0^R d\theta r\psi^*\omega dr = \int_0^R r|\mathbf{v}|^2 dr. \tag{S30}$$

C. Weakly-nonlinear analysis

Here we consider a weakly-nonlinear solutions including only azimuthal modes with $n = 0, \pm1, \pm2$,

$$\psi = C(t)\psi_0(r) + [A_1(t)\exp(i\theta)\psi_1 + A_2(t)\exp(2i\theta)\psi_2 + \text{c.c.}] + w. \tag{S31}$$

Here ψ_0, ψ_1, ψ_2 are the eigenfunctions obtained from linear stability analysis, $C(t), A_{1,2}(t)$ are slowly-varying functions, and w is small correction to the solution. Functions $C(t), A_1(t), A_2(t)$ are obtained from the corresponding orthogonality conditions guaranteeing that w does not grow.

Substituting solution Eq. (S31) into Eq. (S13), and retaining terms for each azimuthal harmonics, we obtain after applying the orthogonality conditions,

$$\partial_t C = \lambda_0 C - c_1C^3 - c_2C|A_1|^2 - c_3C|A_2|^2 - 2c_4\text{Re}A_2A_1^{*2}, \tag{S32}$$
$$\partial_t A_1 = \lambda_1A_1 - b_1A_1|A_1|^2 - b_2A_1C^2 - b_3A_1|A_2|^2 - b_4CA_2A_1^* + \delta_1A_1C + \gamma_1A_2A_1^*, \tag{S33}$$
$$\partial_t A_2 = \lambda_2A_2 - a_1A_2|A_2|^2 - a_2A_2C^2 - a_3A_2|A_1|^2 - a_4CA_1^2 + \delta_2A_2C + \gamma_2A_1^2. \tag{S34}$$

Here $\lambda_{0,1,2}$ are the linear growth rates, and all other coefficients are integrals over the eigenfunctions. In the following, we can consider $A_{1,2}$ complex.

D. Calculations of the nonlinear coefficients

We substitute the approximate solution Eq (S31) into equation (S13) and apply the solvability conditions. The coefficients $m_{0,1,2}$ are the integrals of the eigenmode squares

$$m_{0,1,2} = \int_0^R drr|\mathbf{v}_{0,1,2}(r)|^2, \tag{S35}$$

where the normalized radial eigenmodes are given by Eq. (S18)

$$\psi_n = \frac{1}{\sqrt{m_n}}\left(\frac{G_{n+}}{k_{n+}^2}J_n(k_{n+}r) + \frac{G_{n-}}{k_{n-}^2}J_n(k_{n-}r) + G_{n0}r^n\right). \tag{S36}$$

The nonlinear terms in Eqs. (S32), (S33), (S34) appear from the quadratic term $\mathbf{v}\cdot\nabla\omega$ and the cubic term $\nabla\times|\mathbf{v}|^2\mathbf{v}$.

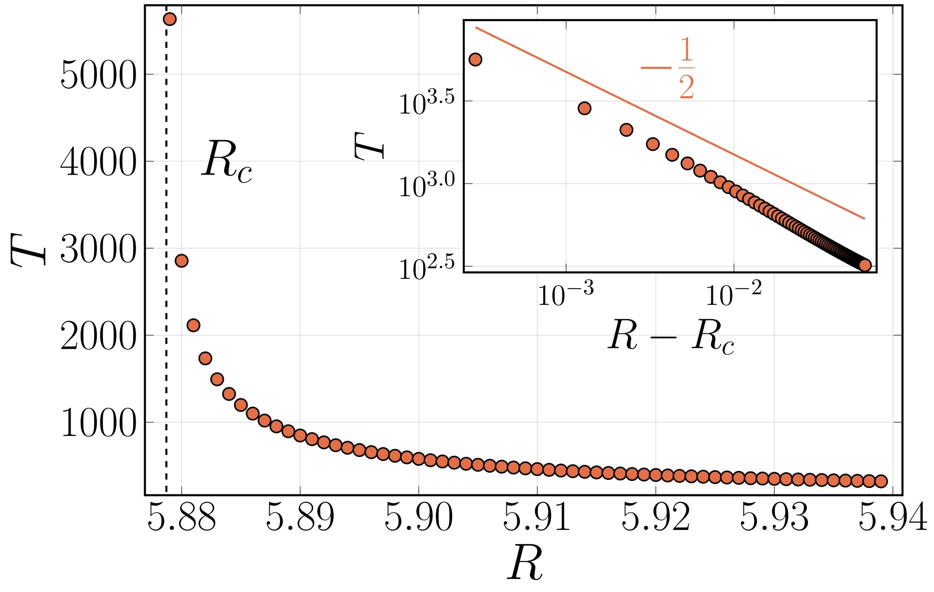

FIG. S10. Period of the limit cycle, obtained from equations (S32), (S33), and (S34), as a function of the well radius R. The dashed line indicates the threshold radius R_c determined by fitting $T \propto (R - R_c)^{-1/2}$, where R_c is the fitting parameter and the best fit gives $R_c = 0.5879$. Inset: Log-log plot of the data. The red solid line represents the slope with the decay exponent $-1/2$ to guide the eye.

The coefficients for the quadratic terms $\delta_{1,2}, \gamma_{1,2}$ are given by the expressions (after partial integration)

$$\delta_1 A_1 C + \gamma_1 A_2 A_1^* = -\frac{\lambda}{2\pi} \int d\theta dr r \mathbf{v}_1^* \exp(-i\theta) \mathbf{v} \cdot \nabla \mathbf{v}, \tag{S37}$$

$$\delta_2 A_2 C + \gamma_2 A_1^2 = -\frac{\lambda}{2\pi} \int d\theta dr r \mathbf{v}_2^* \exp(-2i\theta) \mathbf{v} \cdot \nabla \mathbf{v}. \tag{S38}$$

(S39)

Here $\mathbf{v}_n = (v_{rn}, v_{\theta n}) = (in\psi_n/r, -\partial_r \psi_n)$. Note that in polar coordinates,

$$\mathbf{v} \cdot \nabla \mathbf{v} = (v_r \partial_r v_r + v_\theta \partial_\theta v_r - v_\theta^2/r, v_r \partial_r v_\theta + v_\theta \partial_\theta v_\theta + v_\theta v_r/r). \tag{S40}$$

The advection term does not generate any contribution to Eq. (S32) due to the symmetry.
Correspondingly, for $a_{1,2,3,4}, b_{1,2,3,4}, c_{1,2,3,4}$ we obtain

$$c_1 C^3 + c_2 C|A_1|^2 + c_3 C|A_2|^2 + 2c_4 \mathrm{Re} A_2 A_1^{2*} = \frac{b}{2\pi} \int d\theta dr r \mathbf{v}_0^* \mathbf{v} |\mathbf{v}|^2, \tag{S41}$$

$$b_1 A_1 |A_1|^2 + b_2 A_1 C^2 + b_3 A_1 |A_2|^2 + b_4 C A_2 A_1^* = \frac{b}{2\pi} \int d\theta dr r \mathbf{v}_1^* \exp(-i\theta) \mathbf{v} |\mathbf{v}|^2, \tag{S42}$$

$$a_1 A_2 |A_2|^2 + a_2 A_2 C^2 + a_3 A_2 |A_1|^2 + a_4 C A_1^2 = \frac{b}{2\pi} \int d\theta dr r \mathbf{v}_2^* \exp(-2i\theta) \mathbf{v} |\mathbf{v}|^2. \tag{S43}$$

All linear and nonlinear coefficients are calculated in Mathematica and imported directly into the normal form equations (S32), (S33), (S34), see Mathematica script provided as a Supplementary Material. It appears that $\gamma_1 = \gamma_2$, and $c_4 = a_4 = b_4/2$. The resulting equations generate a limit cycle without any further adjustments. As the radius R decreases, the period of the limit cycle diverges consistently with $T \propto (R - R_c)^{-1/2}$ at the threshold radius $R = R_c$ (Fig. S10). This behavior aligns with the infinite period bifurcation scenario [8].

E. Bridging theory and numerics

To perform faithful comparison, mode decomposition of numerical data was done by spline interpolation on the radial grid followed by the trapezoidal quadrature based on the same formula as in the analytical theory. The upper

bound of r integral was set larger than R (typically $\simeq 9$) to contain the well plus the leakage. The azimuthal modes (Fig. 3(c), Fig. 4(b,c)) were based on the following formulae:

$$v_{*n} = \frac{e^{-i\phi_n}}{2\pi\sqrt{m_n}} \int d\theta\, e^{-in\theta} v_*(r,\theta), \tag{S44}$$

where $* = r, \theta$. $e^{-i\phi_n}$ is introduced to ensure v_{*n} is real-valued and has the consistent sign with the theory. Similarly the associated mode amplitudes (Fig. 3(d), Fig. 4(b)) were calculated by the following:

$$|C| = \sqrt{\frac{\int dr\, r|v_{\theta 0,\mathrm{num.}}|^2}{\int dr\, r|v_{\theta 0,\mathrm{theory}}|^2}}, \tag{S45}$$

$$|A_1| = \sqrt{\frac{\int dr\, r|v_{\theta 1,\mathrm{num.}}|^2}{\int dr\, r|v_{\theta 1,\mathrm{theory}}|^2}}, \tag{S46}$$

$$|A_2| = \sqrt{\frac{\int dr\, r|v_{\theta 2,\mathrm{num.}}|^2}{\int dr\, r|v_{\theta 2,\mathrm{theory}}|^2}}. \tag{S47}$$

As for the sign of C, as $v_{\theta 0,\mathrm{theory}} > 0$, we can naturally identify it as the sign of $\int dr\, v_{\theta 0,\mathrm{num.}}$. Moreover, beyond the sign consistency, the spin variable defined in Eq. (1) is proportional to C, as readily verified: $S_i \propto \iint r\, dr\, d\theta\, r v_\theta = \int dr\, r^2 \int d\theta \left[C v_{\theta 0} + \sum_{n\geq 1} \left(A_n v_{\theta n} e^{in\theta} + \mathrm{c.c.} \right) \right] = C \int dr\, r^2 v_{\theta 0} \propto C$, where A_n are the mode amplitudes and the integrals run over the area of the i-th well. This proportionality was used in Fig. 3(b). With the help of mode decomposition and R_{eff} plotted in Fig. S11(a), we can almost directly compare the theory and simulation for some quantities, such as $\max|\omega|$ shown in Fig. S11(b).

In the large-R region, where we cannot compute reliable estimates of R_{eff} due to oscillation, we still see good agreements between the theory and simulation as in Fig. S11(c,d).

V. SUPPLEMENTARY MOVIE CAPTIONS

Movie 1: Movie of the whole field of view (2.1 mm × 2.1 mm), corresponding to Fig. S1. The initial 20 seconds of the data are presented at twice the real speed (2×). The spatial resolution is lowered to reduce the file size.

Movie 2: The vorticity and velocity fields of the single stabilized vortex ($R = 44.6$ μm). The color bar of the vorticity field is the same as in Fig. 1. The movie is played at real-time speed. The scale bar represents 20 μm.

Movie 3: The vorticity and velocity fields of the reversing vortices ($R = 46.7$ μm). The color bar of the vorticity field is the same as in Fig. 1. The movie is played at real-time speed. The scale bar represents 20 μm.

Movie 4: The vorticity and velocity fields of the four-vortex state ($R = 48.8$ μm). The color bar of the vorticity field is the same as in Fig. 1. The movie is played at real-time speed. The scale bar represents 20 μm.

Movie 5: The vorticity and velocity fields of the turbulent state ($R = 49.5$ μm). The color bar of the vorticity field is the same as in Fig. 1. The movie is played at real-time speed. The scale bar represents 20 μm.

Movie 6: The velocity field of the single stabilized vortex ($R = 44.6$ μm). The velocity vectors are colored based on the sign of the local vorticity: yellow for positive and green for negative. The movie is played at 4× real-time speed. The scale bar represents 20 μm.

Movie 7: The velocity field of the reversing vortices ($R = 46.7$ μm). The velocity vectors are colored based on the sign of the local vorticity: yellow for positive and green for negative. The movie is played at 4× real-time speed. The scale bar represents 20 μm.

Movie 8: The velocity field of the four-vortex state ($R = 48.8$ μm). The velocity vectors are colored based on the sign of the local vorticity: yellow for positive and green for negative. The movie is played at 4× real-time speed. The scale bar represents 20 μm.

Movie 9: The movie of the non-coherent random motion in the small well ($R = 42.9$ μm). The movie is played at real-time speed. The scale bar represents 20 μm.

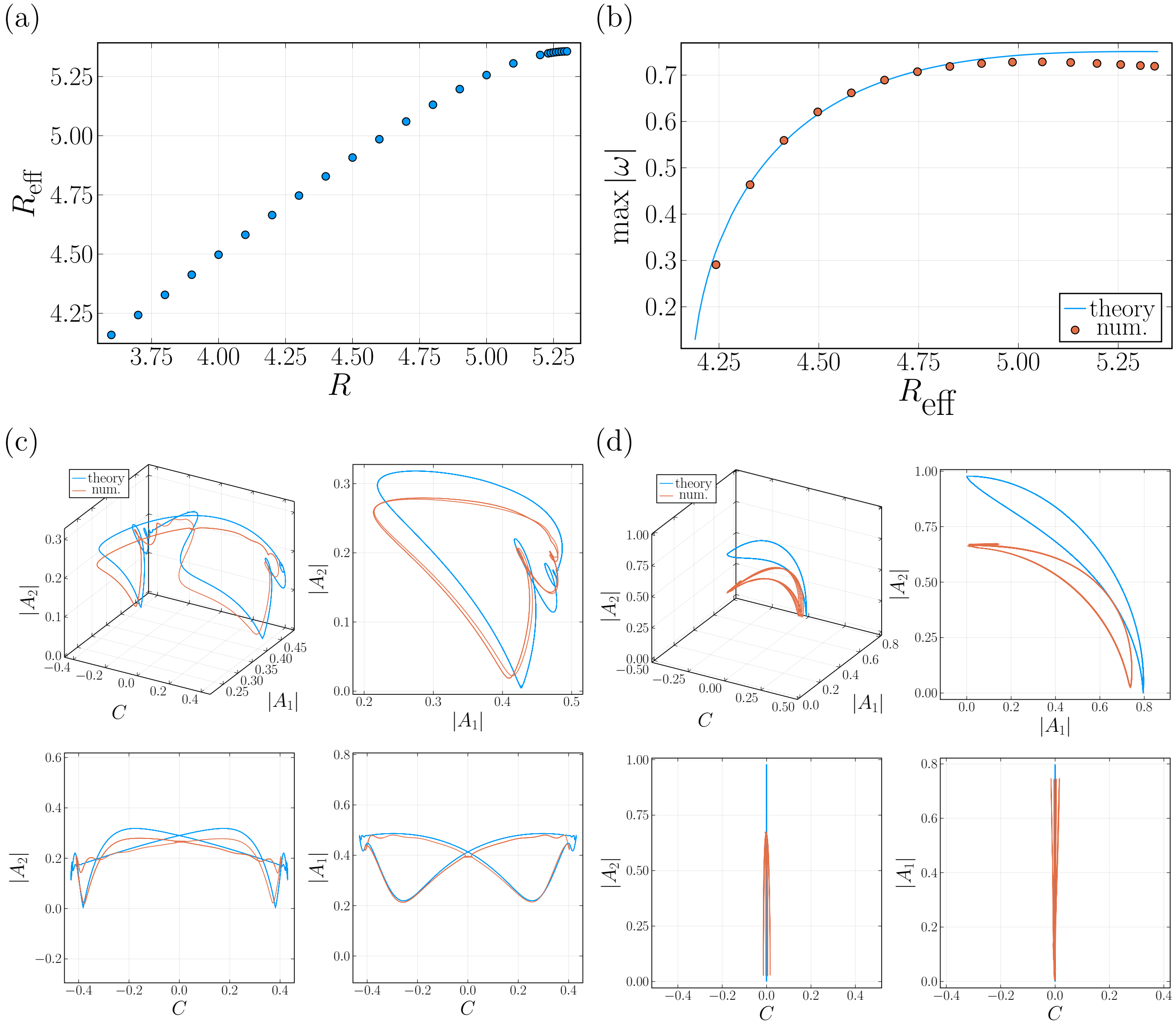

FIG. S11. (a) Radius R used for K (damping mask) and effective radius $R_{\rm eff}$ estimated from the simulation. Generally we have $R_{\rm eff} > R$ due to leakage. (b) R-dependence of max $|\omega|$ near the lower bound radius having $\lambda_0 > 0$. (c,d) Three-dimensional phase space plot and projections, in the two-vortex state ((c), $R = 5.35$ for simulation and $R = 5.9$ for theory) and four-vortex state ((d), $R = 6.4$ for simulation and $R = 7.0$ for theory).

Movie 10: The vorticity and velocity fields of the reversing vortices ($R = 48.0$ μm). The color bar of the vorticity field is the same as in Fig. 1. The movie is played at real-time speed. The scale bar represents 20 μm.

Movie 11: Vorticity field of the single stabilized vortex (Fig. 3(a), left), numerically computed at $R = 5.2$.

Movie 12: Vorticity field of the reversing two-vortex state (Fig. 3(a), the second from the left), numerically computed at $R = 5.6$.

Movie 13: Vorticity field of the pulsating four-vortex state (Fig. 3(a), the third from the left), numerically computed at $R = 6.4$.

Movie 14: Vorticity field of the turbulent state (Fig. 3(a), right), numerically computed at $R = 7.6$.

Movie 15: Vorticity field of the reversing two-vortex state(Fig. 3(b)), numerically computed at $R = 5.35$.

Movie 16: Azimuthal Fourier decomposition of the vorticity field of the reversing two-vortex state at $R = 5.6$ (Fig. 3(c)). The original vorticity field and the modes $n = 0, 1, 2$ are shown.

Movie 17: Vorticity field of the pulsating four-vortex state (Fig. 3(d)), numerically computed at $R = 6.2$.

Movie 18: Vorticity field and its azimuthal Fourier decomposition of the analytically calculated reversing two-vortex state at $R = 5.9$ (Fig. 4(e)). The definitions of the modes are the same as in Supplementary Movie 12 and Fig. 3(c).

Movie 19: Vorticity field and its azimuthal Fourier decomposition of the analytically calculated pulsating four-vortex state at $R = 7.0$ (Fig. 4(f)). The definitions of the modes are the same as in Supplementary Movie 12 and Fig. 3(c).

[1] E. Stamhuis and W. Thielicke, PIVlab – Towards User-friendly, Affordable and Accurate Digital Particle Image Velocimetry in MATLAB, Journal of Open Research Software **2**, 10.5334/jors.bl (2014).

[2] I. A. Beresnev and V. N. Nikolaevskiy, A model for nonlinear seismic waves in a medium with instability, Physica D: Nonlinear Phenomena **66**, 1 (1993).

[3] J. Słomka and J. Dunkel, Spontaneous mirror-symmetry breaking induces inverse energy cascade in 3D active fluids, Proceedings of the National Academy of Sciences **114**, 2119 (2017).

[4] S. L. Brunton, J. L. Proctor, and J. N. Kutz, Discovering governing equations from data by sparse identification of nonlinear dynamical systems, Proceedings of the National Academy of Sciences **113**, 3932 (2016).

[5] H. H. Wensink, J. Dunkel, S. Heidenreich, K. Drescher, R. E. Goldstein, H. Löwen, and J. M. Yeomans, Meso-scale turbulence in living fluids, Proceedings of the National Academy of Sciences of the United States of America **109**, 14308 (2012).

[6] H. Reinken, D. Nishiguchi, S. Heidenreich, A. Sokolov, M. Bär, S. H. Klapp, and I. S. Aranson, Organizing bacterial vortex lattices by periodic obstacle arrays, Communications Physics **3**, 76 (2020).

[7] S. Shiratani, K. A. Takeuchi, and D. Nishiguchi, Route to turbulence via oscillatory states in polar active fluid under confinement, arXiv preprint arXiv:2304.03306 (2023).

[8] S. H. Strogatz, Nonlinear dynamics and chaos: with applications to physics, biology, chemistry, and engineering (studies in nonlinearity), Nonlinear Dynamics and Chaos: With Applications to Physics, Biology, Chemistry, and Engineering (Studies in Nonlinearity) (2001).
